# Intracerebellar injection of monocytic immature myeloid cells prevents the adverse effects caused by stereotactic surgery in a model of cerebellar neurodegeneration

**DOI:** 10.1186/s12974-023-03000-8

**Published:** 2024-02-14

**Authors:** Carlos del Pilar, Lucía Garrido-Matilla, Lucía del Pozo-Filíu, Rafael Lebrón-Galán, Raúl F. Arias, Diego Clemente, José Ramón Alonso, Eduardo Weruaga, David Díaz

**Affiliations:** 1 Institute for Neuroscience of Castile and Leon, INCyL, Universidad de Salamanca, C/Pintor Fernando Gallego 1, 37007 Salamanca, Spain; 2 Institute of Biomedical Research of Salamanca, IBSAL, Salamanca, Spain; 3 Departamento de Psicobiología, Facultad de Psicología, Universidad Nacional de Educación a Distancia (UNED), Madrid, Spain; 4 Translational Stroke Laboratory (TREAT), Clinical Neurosciences Research Laboratory (LINC), Health Research Institute of Santiago de Compostela (IDIS), Santiago de Compostela, Spain; 5 Neuroimmuno-Repair Group, Hospital Nacional de Parapléjicos-SESCAM, Finca La Peraleda s/n, 45004 Toledo, Spain; 6 Centro de Investigación Biomédica en Red de Enfermedades Neurodegenerativas (CIBERNED), Carlos III Health Institute, Av. Monforte de Lemos, 3-5, 28029 Madrid, Spain; 7 Hospital Universitario de Toledo, Avd. Río Guadiana, s/n, 45007 Toledo, Spain

**Keywords:** Selective neurodegeneration, Neuroinflammation, Immune cell modulation, Cell therapy, Surgical brain injury, MDSCs

## Abstract

**Background:**

Myeloid-derived suppressor cells (MDSCs) constitute a recently discovered bone-marrow-derived cell type useful for dealing with neuroinflammatory disorders. However, these cells are only formed during inflammatory conditions from immature myeloid cells (IMCs) that acquire immunosuppressive activity, thus being commonly gathered from diseased animals. Then, to obtain a more clinically feasible source, we characterized IMCs directly derived from healthy bone marrow and proved their potential immunosuppressive activity under pathological conditions in vitro. We then explored their neuroprotective potential in a model of human cerebellar ataxia, the Purkinje Cell Degeneration (PCD) mouse, as it displays a well-defined neurodegenerative and neuroinflammatory process that can be also aggravated by invasive surgeries.

**Methods:**

IMCs were obtained from healthy bone marrow and co-cultured with activated T cells. The proliferation and apoptotic rate of the later were analyzed with Tag-it Violet. For in vivo studies, IMCs were transplanted by stereotactic surgery into the cerebellum of PCD mice. We also used sham-operated animals as controls of the surgical effects, as well as their untreated counterparts. Motor behavior of mice was assessed by rotarod test. The Purkinje cell density was measured by immunohistochemistry and cell death assessed with the TUNEL technique. We also analyzed the microglial phenotype by immunofluorescence and the expression pattern of inflammation-related genes by qPCR. Parametric tests were applied depending on the specific experiment: one or two way ANOVA and Student’s *T* test.

**Results:**

IMCs were proven to effectively acquire immunosuppressive activity under pathological conditions in vitro, thus acting as MDSCs. Concerning in vivo studios, sham-operated PCD mice suffered detrimental effects in motor coordination, Purkinje cell survival and microglial activation. After intracranial administration of IMCs into the cerebellum of PCD mice, no special benefits were detected in the transplanted animals when compared to untreated mice. Nonetheless, this transplant almost completely prevented the impairments caused by the surgery in PCD mice, probably by the modulation of the inflammatory patterns.

**Conclusions:**

Our work comprise two main translational findings: (1) IMCs can be directly used as they behave as MDSCs under pathological conditions, thus avoiding their gathering from diseased subjects; (2) IMCs are promising adjuvants when performing neurosurgery.

**Supplementary Information:**

The online version contains supplementary material available at 10.1186/s12974-023-03000-8.

## Introduction

The regenerative capacity of the central nervous system is limited, which has supposed an enormous challenge for the development of effective therapies to cope with neurodegenerative diseases [1]. Stem cells have emerged as promising agents in an attempt to replace lost neurons or glial cells and for promoting a neuroprotective environment [2–4]. In particular, bone-marrow-derived stem cells constitute one of the most amenable and suitable sources, because they are easily obtained, possess neuroprotective and immunomodulatory properties, and are capable of physiologically integrating into multiple tissue types, including the central nervous system [5–8].

Within the pool of bone-marrow cells, the role of myeloid-derived suppressor cells (MDSCs) in neurodegenerative diseases is gaining importance [9]. MDSCs are a heterogeneous group of immature myeloid cells (IMCs) with highly immunomodulatory properties that originate from IMCs under inflammatory conditions [10, 11]. MDSCs are mainly characterized by their extraordinary ability to inhibit T-cell functions [12, 13]. In addition, they can also modulate glial cells [14] and other myeloid populations, such as dendritic cells and macrophages/microglia [15, 16], the latter being a pivotal player in the acquisition and/or the development of most neurological disorders [17–19]. More precisely, monocytic MDSCs, a subset of MDSCs (see below), divert macrophages/microglia toward a neuroprotective phenotype through cytokines, primarily IL-10 [16, 20, 21].

MDSCs are subdivided into two main groups: monocytic and polymorphonuclear [22, 23]. Both cell subsets control the immune response in a wide manner, mainly through arginase-1, inducible nitric oxide synthase, and by releasing reactive nitrogen species and cytokines [11]. Moreover, it has been shown through immunosuppression assays that monocytic MDSCs have a greater suppressive potential than polymorphonuclear MDSCs, both in vitro and in vivo [23, 24]. Importantly, phenotypic characterization alone is not enough to identify murine MDSCs, since both monocytic and polymorphonuclear subsets possess a phenotype similar to that of the monocytes and neutrophils, respectively. For this reason, functional tests are required when MDSCs are the subject of analysis, especially considering that their potent immunosuppressive activity is what truly distinguishes them from other myeloid cell populations [25, 26].

MDSCs are an excellent tool for examining those inflammatory diseases where controlling the immune response may result in beneficial effects [12]. In fact, the use of MDSCs is currently being explored in the treatment of autoimmune diseases and allotransplants [27, 28]. In the context of neurodegeneration/inflammation, MDSCs have been studied in several pathologies, such as experimental autoimmune encephalomyelitis (EAE), spinal cord injury, strokes, and traumatic brain injury [9, 16]. However, as far as we know, the transplantation of MDSCs has only been conducted in models for EAE and spinal cord injury [6, 9]. Concerning the EAE model, it has been found that transplanted MDSCs delay the onset of symptoms and significantly reduced demyelinated areas [29, 30]. In the case of the spinal cord injury model, it has been observed that the adoptive transfer of monocytic MDSCs in damaged areas significantly attenuates acute inflammation and promotes tissue repair by creating a permissive environment for the development of macrophages with a neuroprotective character [6]. Altogether, MDSCs lead to anti-inflammatory and neurotrophic effects as well as functional improvements. Therefore, MDSC-based therapies may become promising strategies for treating several neurodegenerative disorders that typically progress with neuroinflammation [9].

Traditionally, MDSCs have been isolated from spleen in different murine models of neurodegenerative disorders, as they abundantly accumulate in this organ, but tumor tissue, bone marrow, and even nervous or pulmonary tissue have also been used as viable sources [16, 31, 32]. However, this material depends on the use of diseased individuals, which is undoubtedly problematic if we consider the clinical exploitation of MDSCs. Therefore, for this study, we chose to characterize and transplant monocytic IMCs, as they are the cells from which monocytic MDSCs differentiate [33, 34].

In the present work, we have set out to ascertain whether monocytic IMCs derived from the bone marrow of wild-type (WT) animals can suppress the inflammation/degeneration afflicting a mouse model of cerebellar ataxia: the Purkinje Cell Degeneration (PCD) mutant mouse. This animal model holds a mutation in the *Ccp1* gene (also known as *Agtpbp1* or *Nna1*) that leads to the postnatal loss of specific neuronal populations, such as the Purkinje cells in the cerebellum [35, 36]. More precisely, Purkinje cell loss starts at postnatal day 18 (P18) and progresses quickly in such a way that at P30 these neurons mainly survive in the nodulus (lobe X) and the ventral side of the uvula (lobe IX) [35, 36]. Along with this selective neurodegeneration, an exacerbated reactive gliosis occurs in this region, mainly characterized by hypertrophied microglial cell bodies and the apoptosis of oligodendrocytes [37]. Furthermore, specific alterations in the peripheral immune system have been described [38].

Our findings show how monocytic IMCs effectively exert immunosuppressive activity when cultured under pathological conditions, as well as intriguing anti-inflammatory effects after their intracerebellar transplantation in PCD mice.

## Methods

### Animals

Both WT and PCD mice from the C57BL/DBA hybrid strain were used. These mice were obtained by mating C57BL/6J and DBA/2J strains, both purchased originally from The Jackson Laboratory (Bar Harbor, ME, USA). Transgenic green fluorescent protein (GFP) mice of the BALB/cJ strain (The Jackson Laboratory) were employed as donors of IMCs.

Mice were bred at the Animal Facilities of the University of Salamanca at constant temperature and relative humidity, with a 12/12 h photoperiod, and were fed ad libitum with water and special rodent chow (Rodent toxicology diet, B&K Universal G.J., S.L., Barcelona, Spain). Animal housing and manipulation were carried out in compliance with European (Directive 2010/63/UE, Recommendation 2007/526/CE) and Spanish (Law 32/2007, RD 53/2013) legislation. All the experiments were approved by the Bioethics Committee of the University of Salamanca (reference numbers: #00291 and #00344).

### Study design: animal assignment

For characterizing the IMCs, we used 4 seven-week-old WT mice to isolate T cells and 4 WT mice between the ages of 2 and 3 months to isolate IMCs.

For the intracerebellar injection of IMCs, we used 6 GFP mice as donors and 11 PCD mice as recipients. In addition, we employed 7 PCD mice that had been operated on but without transplantation (sham-operated) and 9 PCD mice that did not receive any treatment. All the donors were sacrificed between 2 and 3 months of age and all the recipients were transplanted at P20. In this study, untreated PCD mice acted as the control (diseased individuals), as intracerebellar injection would not be performed on healthy subjects. In any case, we included two supplemental WT groups, untreated and sham-operated (8 and 7 mice, respectively), to check whether surgery had any possible significant effects on an unharmed system. These experiments were restricted to behavior, apoptosis, Purkinje cell survival and microglial analysis (see below). Briefly, all animals were subjected to rotarod testing at P25 and P30 and were perfused at P30. Five animals per group (three sections per animal) were used for histological analyses. In Table 1, the precise number of animals employed for each experiment can be appreciated.

**Table 1:** Animals used in this work sorted by experiments

IMCs characterization	Behavioral and histological analyses		qPCR analysis
	*Rotarod test (P25 and P30)*	Histological analysis (P30)*	
4 WT to isolate T cells	6 WT GFP (donors)		5 WT
4 WT to isolate IMCs	11 transplanted PCD	5**	6 untreated PCD
	7 sham-operated PCD	5**	4 sham-operated PCD
	9 untreated PCD	5	4 transplanted PCD
	8 untreated WT	5	
	7 sham-operated WT	5	
8	48		19
Total: 75 animals (34 WT and 41 PCD)			

*For histological analysis 3 sections per animal were used

**For the particular analyses of T and B cells, 1 or 2 supplementary animals were added

In addition, we employed 5 WT, 6 untreated PCD, 4 sham-operated PCD, and 4 transplanted PCD mice for genetic qPCR analyses.

In total, 75 mice were used in this study: 34 WT and 41 PCD (Table 1).

### Genotyping

As PCD mice are not suitable for breeding [36], the colony was maintained by mating heterozygous animals, which are indistinguishable from their WT littermates. In consequence, the offspring were genotyped by PCR as previously described [39, 40].

### T-cell isolation and activation

First, spleens were dissected out of WT mice. A single-cell suspension was obtained from spleens by passing the tissue through a 40 µm nylon cell strainer (BD Biosciences, San Jose, CA, USA). Then, red blood cells were lysed in ACK lysis buffer (0.83% w/v NH_4_Cl, 0.1% w/v KHCO_3_, 1 mM EDTA in distilled H_2_O, pH 7.4; Panreac Química, Barcelona, Spain). The resulting splenocytes were resuspended in 200 µl of staining buffer containing 25 mM HEPES, 2% v/v penicillin/streptomycin, and 10% v/v fetal bovine serum (Cultek, Madrid, Spain) in sterile phosphate buffered saline (PBS). Fc receptors were blocked with anti-CD16/CD32 antibodies (10 µg/ml; BD Biosciences) in staining buffer for 10 min at 4 ºC. After blocking, cells were labeled for 30 min at 4 ºC in darkness together with 200 µl of staining buffer with Pacific Blue-conjugated anti-CD3ε antibodies (4 µg/ml; BD Biosciences). Splenocytes were washed twice with staining buffer, recovered by centrifugation at 1,500 rpm for 5 min, resuspended in PBS and, finally, sorted with a FACSAria cell sorter (BD Biosciences). T cells were isolated as CD3^+^ cells.

For T-cell activation, 96-well flat bottom plates were coated with purified anti-CD3ε and anti-CD28 antibodies (1 µg/ml each; BD Biosciences) in PBS for 3 h at 37 ºC. Purified CD3^+^ T cells (2 × 10^5^/well) were plated in RPMI medium (Gibco–Thermo Fisher Scientific, Waltham, MA, USA) supplemented with 25 mM HEPES, 10% v/v fetal bovine serum, 2 mM l-glutamine, 1% v/v penicillin/streptomycin, and 50 µM 2-mercaptoethanol (Sigma-Aldrich, St. Louis, MO, USA), and stimulated for 14 h with anti-CD3ε/CD28, at 37 ºC in 5% CO_2_. Some T cells were seeded with culture medium alone (unstimulated controls).

### IMC isolation and co-culture with T cells

IMCs were isolated from WT mice 1 day after the T cells were plated. First, mice were sacrificed by cervical dislocation, the femurs and tibias were dissected, and bone-marrow cells were extracted by flushing RPMI medium through both epiphyses. After that, the same procedure used to isolate T cells was followed. In this case, we employed the following antibodies: PerCP-Cy5.5-conjugated anti-CD11b (4 µg/ml), FITC-conjugated anti-Ly-6C (10 µg/ml), and PE-conjugated anti-Ly-6G (4 µg/ml; BD Biosciences). IMCs were isolated as Ly-6C^high^/Ly-6G^–/low^ gated on CD11b^+^ cells.

Once sorted, 5 × 10^4^ IMCs were plated with the T cells at a 1:4 ratio, and the co-culture was incubated for 48 h at 37 ºC in 5% CO_2_. IMCs were not seeded in every well, so that some wells only contained T cells. Subsequently, the cells were harvested and both T-cell proliferation and cell cycle were evaluated by flow cytometry.

For the proliferation assay, T cells were previously exposed to 2 µM Tag-it Violet™ Proliferation and Cell Tracking Dye (BioLegend, San Diego, CA, USA) diluted in PBS supplemented with 0.1% w/v bovine serum albumin under continuous rotary shaking for 20 min at 37 ºC and protected from light. For the cell cycle assay, cells were fixed in 70% v/v ethanol at –20 ºC, and stained the following day with a propidium iodide/RNase solution (Immunostep, Salamanca, Spain) according to the manufacturer’s instructions. Then, cells were analyzed in a FACSCanto II cytometer (BD Biosciences) with FACSDiva 6.1 software (BD Biosciences).

### Intracerebellar injection of IMCs

IMCs were extracted as described above from the bone marrow of GFP mice to easily identify them in the recipients thanks to their distinctive fluorescence. Once isolated by flow cytometry, they were transplanted into the cerebellum of PCD mice at P20, specifically into lobes IV–V. To this end, mice were placed in a stereotactic apparatus (Just for Mouse Stereotactic Instrument; Stoelting, Wood Dale, IL, USA) under the effect of inhalational anesthesia (2% v/v isoflurane; Provesa, Murcia, Spain) that was administered through a nose mask (Stoelting). Then, a small incision was made along the midline of the back of the head to expose the skull. Next, a small burr hole was made 2.2 mm caudal from lambda in the midline. Subsequently, IMCs (1.3 × 10^5^ in 2 µl PBS) were injected using a Hamilton 75N syringe with a 26G needle (Hamilton, Reno, NV, USA). The needle was inserted 2.5 mm with respect to the bone surface the bone surface and the cell suspension was inoculated at a rate of 0.5 µl/min. Sham-operated animals (either PCD or WT for the supplementary study) underwent the same transplantation procedure, but only the vehicle was injected (2 µl PBS). The needle was left in place for 2 min before it was withdrawn. Then, the scalp was sutured, and each animal was subcutaneously administered a dose of 2 mg meloxicam/kg body weight (Metacam; Boehringer Ingelheim, Ingelheim am Rhein, Germany) and 0.1 mg buprenorphine/kg body weight (Buprecare; Divasa-Farmavic, S.A., Barcelona, Spain) for postoperative pain relief. Finally, each mouse was kept on a heating pad until recovering from the anesthesia and then transferred to a standard cage.

### Rotarod performance test

The 3 groups of PCD animals (untreated, transplanted, and sham-operated) were subjected to the rotarod test (Rotarod LE8200; Panlab, Barcelona, Spain) at P25 and P30 to evaluate their motor coordination and balance. The test consisted of positioning each animal on a horizontal rod rotating at constant acceleration (from 4 to 40 rpm in 10 min) and measuring the time the animal remained on the rod. In each session, seven trials were conducted with a 15-min break between each one to prevent the animals from becoming fatigued. The latency to fall (s) was automatically recorded. The apparatus was cleaned with 96% v/v ethanol between trials. For the statistical analysis, the longest duration out of the seven measurements taken was considered the value that best represented the animal’s motor skills. This test was repeated with the two supplementary groups of WT mice (untreated and sham-operated).

### Tissue preparation

At P30, the mice were deeply anesthetized and subsequently perfused intracardially with 0.9% w/v NaCl for 1 min, followed by Somogyi’s fixative solution containing 4% w/v paraformaldehyde and 15% v/v saturated picric acid in 0.1 M phosphate buffer (PB) for 15 min. Brains were dissected out and immersed in the same fixative for 2 h at room temperature. Then, they were rinsed with PB and cryoprotected with 30% w/v sucrose in PB overnight at 4 °C. Afterward, cerebella were cut in 40-µm sagittal sections by employing a freezing–sliding microtome (Jung SM 2000, Leica Instruments, Nussloch, Germany). The sections collected were rinsed with PB to remove fixative and sucrose residues and immunostained to perform the histological analyses (five animals per group).

For qPCR analyses, mice were deeply anesthetized and then decapitated at P30. Brains were freshly extracted with RNase-free material and cerebella were dissected. Then, the vermis of each cerebellum was separated from the hemispheres. The tissue was immediately snap frozen with liquid nitrogen and stored at -80 ºC until used.

### Hematoxylin and eosin staining

Standard hematoxylin and eosin (H&E) staining was performed to determine the presence of morphological abnormalities, necrotic areas, leukocyte infiltration or microbleedings in the injection site. Due to the thickness of the sections, we have immersed the tissue during 2 min into the hematoxylin solution and during 1 min into the eosin solution, to obtain the most suitable staining.

### Immunofluorescent labeling

Free-floating sections were washed with PBS (3 × 10 min) and incubated for 72 h at 4 °C under continuous rotary shaking in a medium containing 0.2% v/v Triton X-100, 5% v/v normal donkey serum, and the primary antibodies: mouse anti-calbindin D-28 k (CB; 1:2000; 300, Swant, Marly, Switzerland), rabbit anti-CD3 (1:500; ab16669, Abcam, Cambridge, UK), rat anti-CD16/CD32 (1:200; 553142, BD Biosciences), rat anti-CD45 (1:1000; MCA1388, Bio-Rad Laboratories, Hercules, CA, USA), rat anti-CD45R/B220 (1:200; 553089, BD Biosciences), goat anti-CD206 (1:200; AF2535, R&D Systems, Minneapolis, MN, USA), goat anti-GFP (1:2000; ab5450, Abcam) or rabbit anti-Iba1 (1:1000; 019–19741, Wako Pure Chemical Industries, Ltd., Osaka, Japan) in PBS. These antibodies were used to stain Purkinje cells (CB^+^), T cells (CD3^+^), reactive microglia (Iba1^+^/CD45^+^), B cells (CD45R/B220^+^), pro-inflammatory microglia (Iba1^+^/CD16/CD32^+^), anti-inflammatory microglia (Iba1^+^/CD206^+^), and the transplanted cells (GFP^+^). Following this, sections were rinsed with PBS (3 × 10 min) and then incubated in a second medium for 1 h and 30 min at room temperature under continuous rotary shaking. This second medium contained the appropriate secondary antibodies conjugated to Cy2 or Cy3 (1:500; Jackson ImmunoResearch Laboratories, Cambridge, UK) in PBS. The only exception was the immunofluorescence comprising the antibody against CD45R/B220, which was directly conjugated with phycoerythrin, thus the second medium being not necessary. Ten minutes prior to the end of the incubation, DAPI (4′,6-diamidino-2-phenylindole; Sigma-Aldrich) at 1:10,000 was added to the medium to obtain a nuclear counterstain. Finally, the sections were rinsed with PBS, mounted on gelatin-coated slides, and covered using a freshly prepared anti-fading mounting medium. Appropriate negative controls without the primary antibodies were performed and no staining was observed in any case.

### TUNEL assay

The terminal deoxynucleotidyl transferase-mediated dUTP–biotin nick end labeling (TUNEL) technique was employed to detect apoptotic cells, as previously described [40]. Tissue slices were washed with PBS (3 × 10 min) and treated with a cold solution of ethanol–acetic acid (2:1) for 5 min. Then, slices were washed with PBS (3 × 10 min) and permeabilized with 0.2% v/v Triton X-100 and 0.1% w/v sodium citrate in distilled water for 15 min. They were again washed with PBS (2 × 10 min) and incubated with TUNEL buffer containing 30 mM Tris–HCl, 140 mM sodium cacodylate, 1 mM cobalt(II) chloride, and 0.3% v/v Triton X-100 for 30 min. Subsequently, the tissue slices were incubated in a medium with terminal transferase (800 U/ml; Roche, Basel, Switzerland) and biotinylated dUTP (1 µM; Roche) in TUNEL buffer for 1 h and 30 min at 37 ºC. The reaction was terminated by adding saline sodium citrate buffer, composed of 150 mM sodium chloride and 15 mM sodium citrate in distilled water. Finally, the slices were washed with PBS (3 × 10 min) and developed with a medium containing Cy3-conjugated streptavidin (1:200; Jackson ImmunoResearch Laboratories) in PBS for 1 h and 30 min. Both nuclear counterstaining and slide mounting were performed as described above.

### RNA isolation, reverse transcription, and quantitative PCR analyses

To test whether surgery and cell transplantation modified the expression of neuroinflammation-related genes (Table 2), the real-time quantitative PCR (RT-qPCR) technique was carried out. For this purpose, the freshly extracted vermis were homogenized using an Ultra-Turrax disperser (IKA, Staufen, Germany) and total cytoplasmic RNA was isolated and purified using the column-based PureLink™ RNA Mini Kit (Invitrogen, Thermo Fisher Scientific, Waltham, MA, USA) and the PureLink™ DNase Set (Invitrogen, Thermo Fisher Scientific). From each sample, 1000 ng of total RNA were converted to cDNA using the High-Capacity cDNA Reverse Transcription Kit (Applied Biosystems, Foster City, CA, USA). Quantitative changes in mRNA were measured by RT-qPCR using the corresponding cDNA, PowerUp SYBR Green Master Mix (Applied Biosystems), and the specific pairs of primers listed in Table 2. RT-qPCR assays were carried out with the amplification cycling conditions described in the manufacturer’s protocol for the QuantStudio™ 7 Flex RT PCR System (Applied Biosystems). Three replicates of each biological sample were analyzed, and *Gapdh* was used as an endogenous control to normalize the data. The relative gene expression was calculated as the fold change expression using the normalized Cycles to Threshold (Ct) values. Standard and melting curve analyses were performed for all primers to ensure the efficiency and specificity of the amplification of the genes of interest.

**Table 2:** Primers for qPCR used in this work

Gene targeted	Forward sequence	Reverse sequence
*Cox2*	GGTCATTGGTGGAGAGGTGTA	TGAGTCTGCTGGTTTGGAATAG
*Gapdh*	GCCTATGTGGCCTCCAAGGA	GTGTTGGGTGCCCCTAGTTG
*Ifn-γ*	CAGCAACAGCAAGGCGAAAAAGG	TTTCCGCTTCCTGAGGCTGGAT
*Il-1β*	TGCTCATGTCCTCATCCTGGAAGG	TCGCAGCAGCACATCAACAAGAG
*Il-6*	GAGGATACCACTCCCAACAGACC	AAGTGCATCATCGTTGTTCATACA
*iNos*	CTTTGCCACGGACGAGAC	AACTTCCAGTCATTGTACTCTGAGG
*NfκB*	GCTGCCAAAGAAGGACACGACA	GGCAGGCTATTGCTCATCACAG
*Tfn-α*	GCTTGTCACTCGAATTTTGAGA	ATGTCTCAGCCTCTTCTCATTC

### Microscopy visualization and cell counting

Sections were observed under an epifluorescence microscope Olympus Provis AX70 equipped with an Olympus DP70 digital camera (12.5 MP, Olympus, Tokyo, Japan), which also allows bright field visualization, or a STELLARIS 8 confocal microscope (Leica Microsystems). Epifluorescence images were taken with the 2X, 20X or 40X objectives. Bright field images were taken with the 2X or 20X objectives. Confocal images were taken with the 20X objective.

Digital images were processed using Adobe Photoshop CC 2015 (Adobe Inc., San Jose, CA, USA) to slightly adjust contrast, brightness, and color balance.

For the cerebellar histological analyses, three sections of vermis per animal (five animals per group, see Table 1) were chosen, where all cerebellar lobes could be clearly seen and the degeneration occurs early on [36]. Transplanted cells, Purkinje cells and apoptotic cells were manually counted during the examination of all the sections. Transplanted cells were counted as GFP^+^ cells taking all the sections affected by the injection and the two adjacent sections. Purkinje cell linear density was calculated as the number of CB^+^ cells per mm of the Purkinje cell layer in each section, on one hand, respecting the whole vermis and, on the other hand, respecting the lobe X, which is the region, where most Purkinje cells survive at P30 [36, 41]. Apoptotic cell density was calculated as the number of TUNEL^+^ cells per mm^2^ both over the whole vermis and separately in lobes IV–V, where the cell suspension was injected.

Microglial cells positive for Iba1 were counted in different representative areas: (a) lobes IV and V, which received the transplantation; (b) lobe X, where Purkinje cells resist longer until approximately P40; and c) lobe VIII, which was selected as a conventional region far away from the other areas. In this case, one image was taken per representative area. Three types of microglial cells were evaluated depending on the marker that co-localized with Iba1: CD45^+^ (reactive), CD16/CD32^+^ (pro-inflammatory), or CD206^+^ (anti-inflammatory). The density of these three microglial populations was calculated per mm^2^. All data were analyzed using Neurolucida (V8.23), Neuroexplorer (MBF Bioscience, Williston, VT, USA), and ImageJ (NIH, Bethesda, MD, USA) software. Each count was performed by the same person and following the same criteria.

Here it is necessary to note that some infiltrated peripheral leukocytes may be misinterpreted as Iba1^+^ microglial cells. These cells display a round or rod-shaped morphology, also being negatives for other microglial markers, such as TMEM119, and they have been previously typified in our mouse model [38]. Such cells were not considered in our study, only including the branched or amoeboid Iba1^+^ elements that correspond to pure microglial cells.

### Statistical analysis

All data are shown as mean ± standard error of the mean (SEM). The normality of samples was checked using the Kolmogorov–Smirnov test, which allowed employing parametrical tests in almost all studies. Only the data of microglia analysis did not fit with normality, owing to the high level of data variability of some of the experimental groups. Then, to maintain coherence with the rest of the analyses, data were converted to their Ln [42, 43] they becoming normal, and parametrical tests could be performed (see below).

For the rotarod test, a repeated measures ANOVA test was used to compare the behavior among either the three groups of PCD mice (untreated, transplanted, and sham-operated) or between the additional WT groups at different ages (P25 and P30). When this first analysis resulted significant and once checked that there was no interaction between age and treatment, we performed either the one-way ANOVA and Bonferroni post hoc tests or the Student’s *T* test to assess for differences among the PCD mice or between the supplementary WT mice, respectively, for each age group.

For the histological analyses of Purkinje cell density and microglial reaction, a two-way ANOVA test was carried out to check for possible differences among the experimental groups and the areas analyzed. When statistical differences were detected, either the one-way ANOVA followed by the Bonferroni post hoc test or the Student’s *T* test (when corresponded, depending on the number of groups to be compared) were also carried out to compare the different experimental groups. The analysis of Purkinje cell density in the different groups of PCD mice revealed the only exception, because the differences between lobe X and the rest of the vermis were evident [41]. Thus, the two-way ANOVA test had not sense to be applied: it would report an interaction between factors promoted by regions that statistically behave distinctly (lobe X and the rest of vermis).

For histological analysis of apoptosis and for gene analyses, the one-way ANOVA with the subsequent Bonferroni post hoc tests or the Student’s *T* test (when corresponded) were employed.

All analyses were performed using the IBM SPSS Statistical 25 software (IBM, Armonk, NY, USA).

## Results

### Functional characterization of bone-marrow-derived IMCs

First, we ascertained whether IMCs isolated from healthy bone marrow could acquire immunosuppressive properties under pathological conditions, i.e., if these cells become genuine MDSCs. For this purpose, bone-marrow-derived IMCs were cultured in the presence of both activated and unstimulated (control) T cells to evaluate their immunosuppressive activity. Two days after co-culturing, the cells were harvested and both the proliferative capability and the cell cycle of the T cells were analyzed by flow cytometry.

**Fig. 1:**
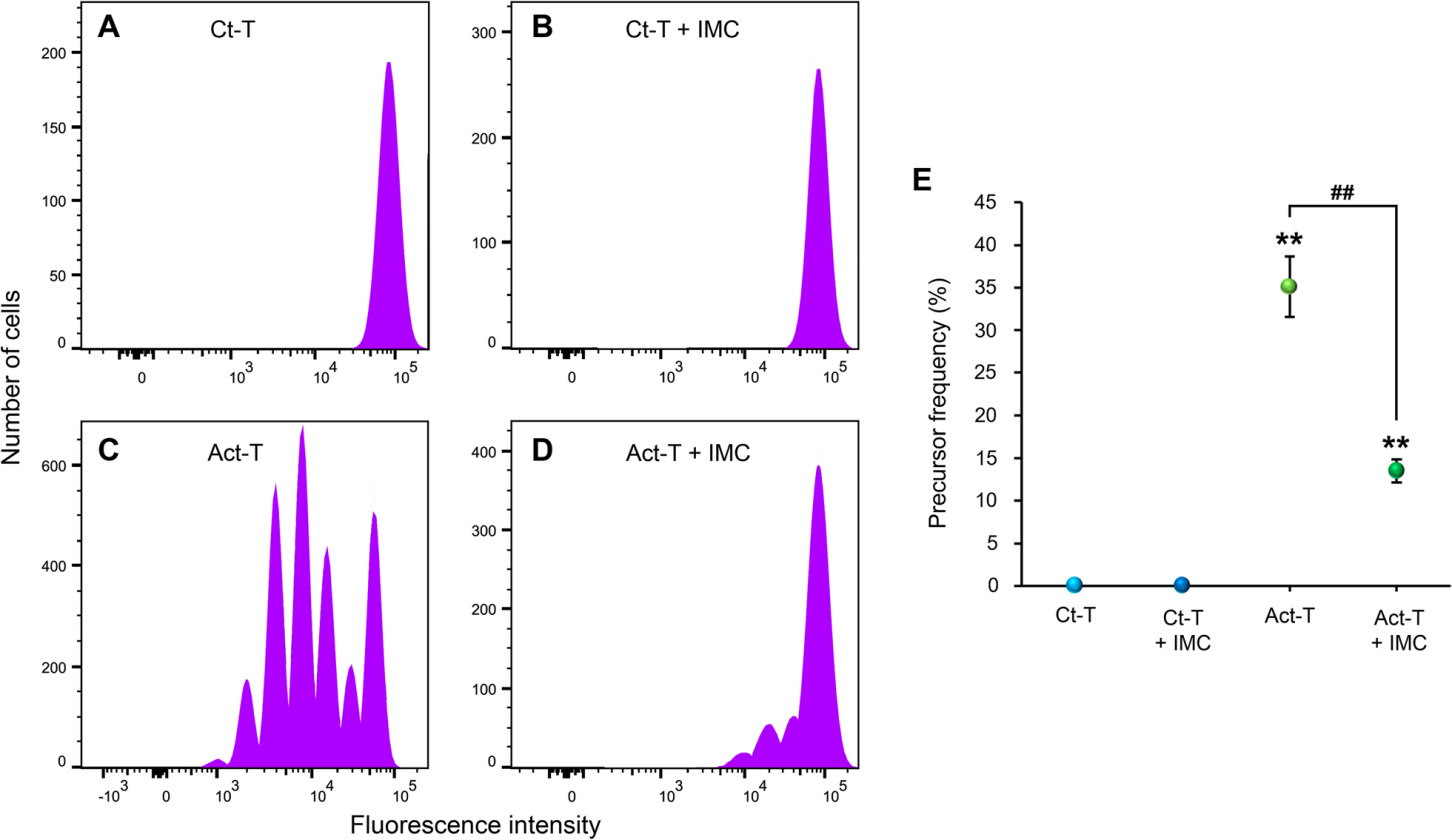
Proliferation analysis of T cells after being cultured with IMCs or alone. **A**–**D** Representative histograms obtained from the flow cytometry analysis of control T cells alone (**A**), control T cells plus IMCs (**B**), activated T cells alone (**C**), and activated T cells plus IMCs (**D**). **E** Chart showing the precursor frequency for the four aforementioned conditions in **A**–**D**. Note how when cultured with IMCs there is a considerable reduction in the proliferation of activated T cells. Data are expressed as mean ± SEM. **p < 0.01, regarding control T cells alone; ^##^*p* < 0.01, effect of IMCs on activated T cells. *Ct-T* control T cells, *Act-T*-activated T cells

Proliferation was assessed by labeling the T cells with Tag-it Violet, a fluorophore with a fluorescent intensity that is inversely proportional to the percentage of dividing cells. The “precursor frequency” was the variable used to compare the different experimental groups. This variable represents the fraction of the original population that divides at least once during the culture period and, importantly, does not depend on the number of cells proliferating or the culture time [44]. Control T cells cultured alone or together with IMCs did not proliferate (Fig. 1A, B). By contrast, activated T cells divided considerably (Fig. 1C). However, when the activated T cells were cultured with IMCs, the percentage of dividing cells substantially decreased (Fig. 1D). The statistical analysis of the activated T cells revealed how the precursor frequency was notably reduced when they were cultured in combination with IMCs (*p* < 0.001; Fig. 1E). Hence, IMCs were able to suppress T-cell proliferation.

**Fig. 2:**
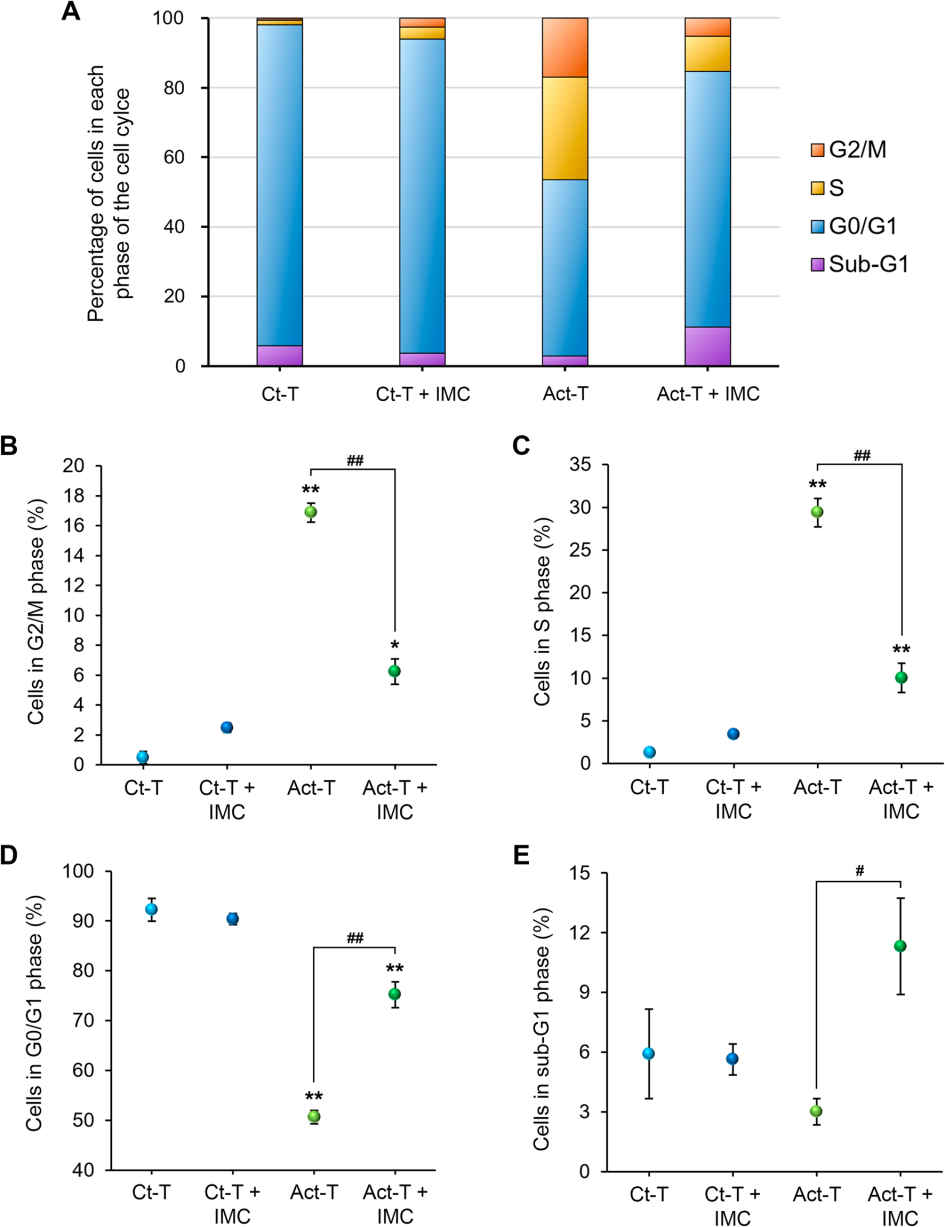
Cell cycle analysis of T cells after being cultured with IMCs or alone. **A** Bar chart representing the percentage of T cells in each phase of the cell cycle in the different experimental groups. **B**–**E** Charts showing the statistical information represented in A separately: G2/M phase (**B**), S phase (**C**), G0/G1 phase (**D**), and sub-G1 phase (**E**). Note how IMCs not only reduce the proliferation of T cells but also induce their apoptosis. Data are expressed as mean ± SEM. *p < 0.05, **p < 0.01, regarding control T cells alone; ^#^*p* < 0.05, ^##^*p* < 0.01, effect of IMCs on activated T cells. *Ct-T* control T cells, *Act-T*-activated T cells

In addition, we assessed the proportion of T cells in each phase of the cell cycle. To this end, their DNA was stained with propidium iodide, a fluorescent intercalating agent. An overview of these results can be appreciated in Fig. 2A and the statistical analyses in Fig. 2B–E. In the group of control T cells, the G0/G1 phase was clearly predominant, indicating there was virtually no proliferation. Conversely, in the group of activated T cells, the percentage of cells in G0/G1 diminished, while, in parallel, both S and G2/M phases augmented, indicating that the cells were dividing. Finally, when activated T cells were cultured with IMCs, the proportion of cells in both S and G2/M phases decreased, showing a reduction in the T-cell proliferative rate. All of this information corroborates the outcomes observed from the proliferation assay. In addition, and more interestingly, we could identify the percentage of cells in apoptosis (sub-G1 phase). In this regard, an increase in the sub-G1 phase was observed when activated T cells were cultured with IMCs (*p* = 0.031; Fig. 2E), which was not observed in co-cultures containing the control T cells (p = 1.000; Fig. 2E). This finding indicates that IMCs can induce T-cell apoptosis.

After having checked the immunosuppressive properties of IMCs under pathological conditions, we proceeded to transplant the cells into the cerebellum of PCD mice at P20, when the degeneration of Purkinje cells had already begun. Both transplanted and sham-operated animals were subjected to stereotactic surgery. In addition, another group of non-treated PCD mice was used to compare the results.

### Motor behavior analysis

**Fig. 3:**
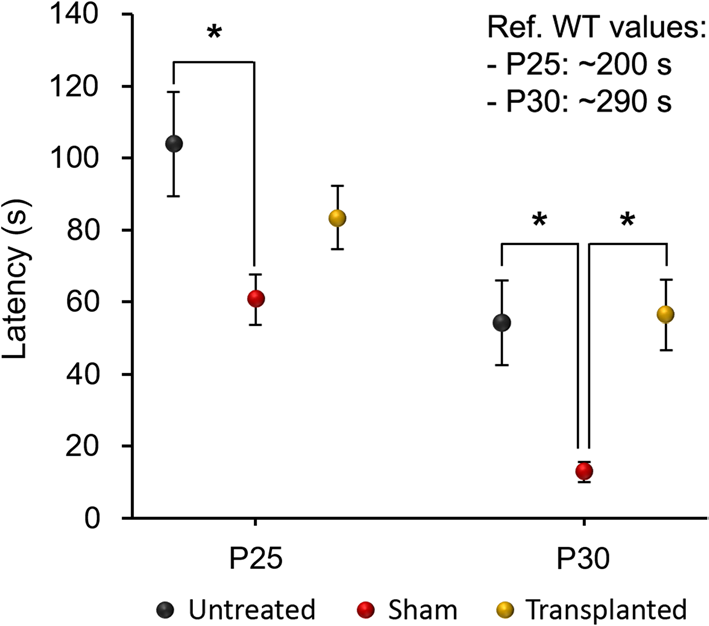
Analysis of the motor coordination of PCD mice. Latency to fall (s) was measured in untreated, transplanted, and sham-operated mice at P25 and P30. Note how the sham-operated animals exhibit a general decrease in their motor coordination, which was avoided by transplanting IMCs. Ref., reference. Data are expressed as mean ± SEM. *p < 0.05

The animals’ motor skills were assessed using the rotarod test (see Material and Methods). After performing ANOVA for repeated measures, we detected differences among the experimental groups (*p* = 0.006), reflecting an effect of treatments, and between ages (*p* = 0.000). These results indicated that motor performance worsened as cerebellar degeneration progressed (Fig. 3). Moreover, an interaction between both factors was also detected (*p* = 0.007; Fig. 3). Again, this indicated that the poor performance in executing the rotarod test differed over time depending on the experimental group. Then, we compared the different experimental groups within each age, revealing interesting data. The performance of the sham-operated animals was worse, both at P25 (*p* = 0.046) and at P30 (*p* = 0.029), as compared to the untreated PCD animals (Fig. 3). However, this alteration was not observed in transplanted PCD mice (*p* = 0.494 at P25 and *p* = 1.000 at P30; Fig. 3), which behaved in a similar way to the untreated animals. Moreover, at P30 the transplanted animals behaved differently to the sham-operated animals (*p* = 0.016; Fig. 3). These findings suggest that although the intracerebellar injection of IMCs did not improve the motor skills of PCD mice, they appeared to effectively protect the animals from the harmful effects resulting from surgery.

Concerning the supplementary WT groups, ANOVA for repeated measures revealed the absence of an interaction between treatment and age (*p* = 0.702) and no differences among the factors analyzed individually (age,* p* = 0.116; treatment, *p* = 0.244; Additional file 1: Fig. S1A). Hence, surgery and stereotactic injection seem not to have any evident effect on movement when the cerebellum is healthy.

### Overall injury assessment

After examining H&E stained sections, we did not observe special abnormalities in the proximity of the injection site, such as an exacerbated leukocyte infiltration, necrotic areas or microbleedings, apart from the consequent destruction of the cerebellar cortex (Additional file 2: Fig. S2).

### IMC integration

**Fig. 4:**
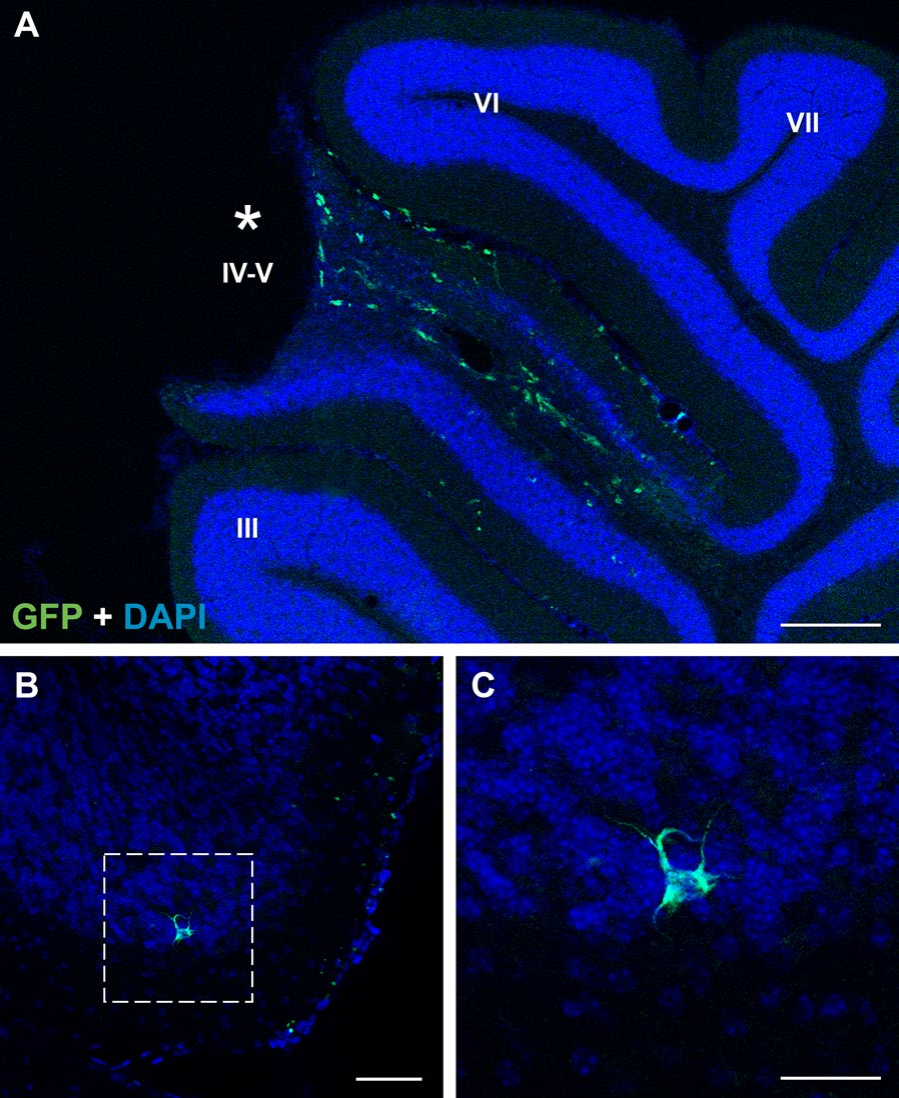
Identification of transplanted cells in the cerebellum of recipient animals at P30. **A** Immunolabeling of GFP^+^ IMCs (green) in the cerebellum of one transplanted PCD mouse; note how transplanted cells are located mainly in lobes IV–V, where the injection was performed (asterisk). **B** Immunolabeling of a single GFP^+^ cell (green) at a distance from the injection site. **C** Magnification of the outlined area in B; note the ramified morphology. Nuclei were counterstained with DAPI (blue). Scale bar: 200 μm (**A**), 50 μm (**B**), 25 μm (**C**)

The next analysis consisted of examining the integration of cells derived from the transplant into the cerebella of the recipients. Consequently, GFP^+^ cells, with a rounded or rod-shaped morphology, were mainly observed in lobes IV–V, which had received the injection (Fig. 4A). More distant and isolated cells were also detected with some ramifications (Fig. 4B, C). We were able to detect transplanted cells in all the sections that presented a surgical damage in the lobes IV–V, which were around 12 in each animal subjected to IMC transplant. This number of sections coincide with the size of the Hamilton’s 26G needle (463.6 µm of outer diameter) and the thickness of the sections of this work (40 µm/section) by doing calculations:

$$\frac{463.6\mathrm{ \ \mu m }}{40\ \mathrm{ \mu m}/{\text{section}}} =11.59\ {\text{sections}}\ (\approx 12\ {\text{sections}})$$

In addition, we have detected transplanted cells only in the two adjacent sections to those presenting a needle damage. Then, the lateral spreading of injected cells 10 days after injury was not higher than 40 µm (with the exception of the aforementioned distant isolated cells). In sum, we were able to detect the majority of transplanted cells in around 14 sections (12 with damage + 2 additional) for each transplanted animal. After conducting a quantification, we identified a mean of 92.50 ± 19.11 cells in each section. Therefore, we estimated a total number of 1257.08 ± 259.68 cells in each transplanted animal (92.50 ± 19.11 cells/section × 11.59 sections). That is to say, we found that approximately 1% of the transplanted cells were still present in the cerebellar vermis 10 day post-transplantation.

Therefore, IMCs could be detected at least 10 days after their transplantation, without showing high migration or survival rates.

### Graft rejection assessment

Since grafts can exert rejection reactions, usually driven by lymphocytes, we quantified the number of T and B cells in the vermis of the three experimental groups, considering either the lobes IV–V (lesion/injection), VIII (neutral) or X (neuroresistant). In both cases, the number of T (Table 3) and B cells (Table 4) was very scarce in all experimental groups, especially in lobes VIII and X. Only in the lobes IV–V of some experimental subjects corresponding to either sham or transplanted PCD mice, 5 or more T cells could be detected. Therefore, only a possible non-generalized surgery effect may exist, and no evident immune rejection reaction seems to be present, which corresponds to the immunomodulatory capability of MDSCs (see Discussion).

**Table 3:** CD3^+^ cells (T cells) in different cerebellar lobes for each experimental condition

Animal	Exp group	Evident lesion	Presence of transplanted cells	CD3^+^ cells lobes IV–V (injection)	CD3^+^ cells lobe VIII (neutral)	CD3^+^ cells lobe X (resistant)
18,126	PCD	N/A	N/A	0	0	0
18,127	PCD	N/A	N/A	3	1	2
18,128	PCD	N/A	N/A	2	0	1
18,171	PCD	N/A	N/A	2	0	1
18,222	PCD	N/A	N/A	4	1	1
18,120	Sham	No	N/A	4	3	1
18,198	Sham	No	N/A	**5**	0	1
18,200	Sham	Yes	N/A	1	2	1
18,201	Sham	Yes (small)	N/A	**6**	1	2
18,202	Sham	Yes	N/A	1	1	1
18,209	Sham	No	N/A	0	0	1
19,026	Sham	No	N/A	0	0	0
18,208	Transp	No	Few	2	0	2
18,237	Transp	No	Yes	1	2	2
18,238	Transp	Yes (big)	Yes	**14**	3	0
18,239	Transp	Yes (small)	Yes	3	1	0
18,241	Transp	No	Yes	0	0	1
19,025	Transp	No	Few	**5**	0	3
19,029	Transp	Yes	Few	**14**	0	1

Values are highlighted (Bold), where 5 or more cells were detected. *Exp* experimental, *N/A* not applicable, *transp* transplanted

**Table 4:** CD45R/B220^+^ cells (B cells) in different cerebellar lobes for each experimental condition

Animal	Exp group	Evident lesion	Presence of transplanted cells	CD45R/B220^+^ cells lobes IV–V (injection)	CD45R/B220^+^ cells lobe VIII (neutral)	CD45R/B220^+^ cells lobe X (resistant)
18,126	PCD	N/A	N/A	0	0	1
18,127	PCD	N/A	N/A	1	1	0
18,128	PCD	N/A	N/A	0	0	2
18,171	PCD	N/A	N/A	3	2	1
18,222	PCD	N/A	N/A	2	0	0
18,120	Sham	No	N/A	1	0	0
18,198	Sham	Yes	N/A	0	0	0
18,201	Sham	No	N/A	2	0	0
18,209	Sham	Yes	N/A	2	0	0
19,026	Sham	No	N/A	1	1	1
19,199	Sham	Yes (small)	N/A	1	0	0
18,208	Transp	No	Scarce	1	0	0
18,237	Transp	Yes (small)	Yes	2	0	3
18,238	Transp	Yes	Yes	3	0	0
18,239	Transp	No	Yes	1	0	1
18,241	Transp	No	Scarce	0	0	0
19,025	Transp	Yes (small)	Yes	0	0	0
19,029	Transp	Yes	Yes	3	0	0

*Exp* experimental, *N/A* not applicable, *transp* transplanted

### Apoptosis assay

**Fig. 5:**
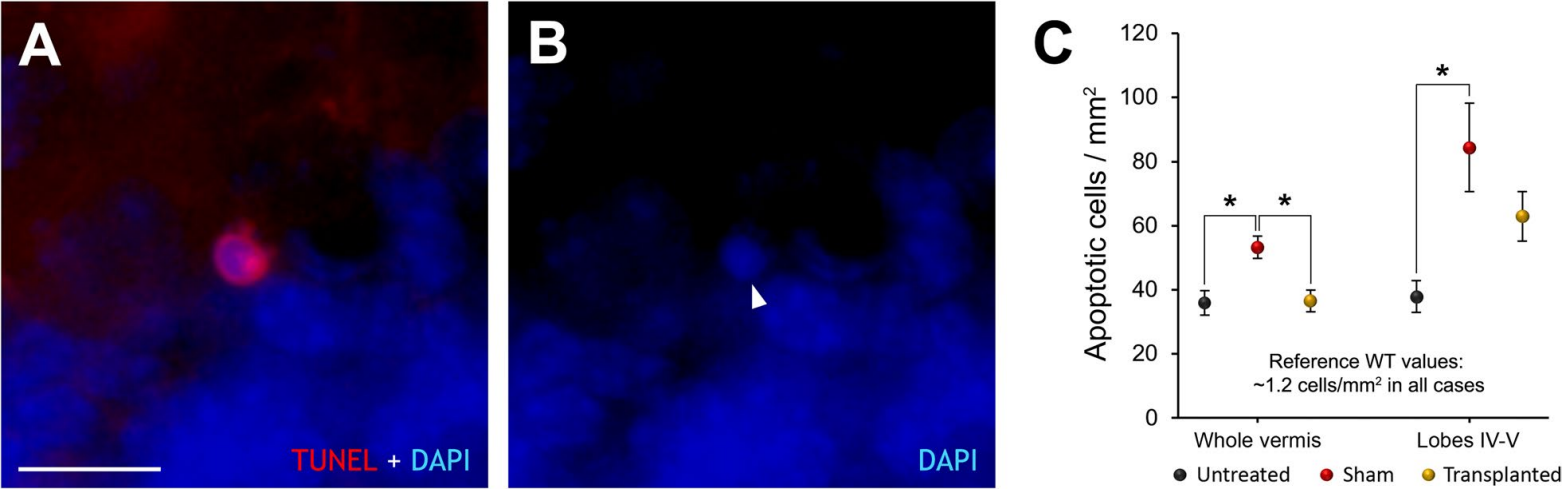
Analysis of cell death. **A**, **B** Apoptotic cell labeled using the TUNEL technique (red); note the condensed chromatin (arrowhead) labeled with DAPI (blue). **C** Chart showing the density of apoptotic cells in untreated, transplanted, and sham-operated PCD animals; note how sham-operated animals present a greater density of apoptotic cells, as well as the protective effect achieved by transplanting IMCs. Data are expressed as mean ± SEM. *p < 0.05. Scale bar: 25 μm

To look for the effects of transplantation, the density of apoptotic cells was first determined using the TUNEL technique. Considering the whole vermis, sham-operated animals had more apoptotic cells than untreated (*p* = 0.014) mice. This increased cell death, however, was prevented in the transplanted mice (*p* = 0.018; Fig. 5), which had a similar level of apoptotic cells as their untreated counterparts (*p* = 1.000; Fig. 5). We also identified, in lobes IV–V (the injection site), an increased density of programmed cell death in the sham-operated animals as compared with untreated mice (*p* = 0.015; Fig. 5). In this analysis, transplanted mice had an intermediate level of apoptotic cells as compared to the untreated and sham-operated mice, and no statistically significant differences were detected among them (Fig. 5). These findings show that the increased apoptosis induced by stereotactic surgery in sham-operated mice was absent in IMC transplanted mice when compared to the other two groups.

In the case of the supplementary WT mice, we also detected increased cell death in the whole vermis (*p* = 0.038) but specifically in the injected lobes (IV–V; *p* = 0.04; Additional file 1: Fig. S1B). These results suggest that stereotactic surgery induces a generalized apoptosis in the vermis of WT mice as well as in PCD animals, although without triggering behavioral effects in the former (see above).

### Purkinje cell survival

**Fig. 6:**
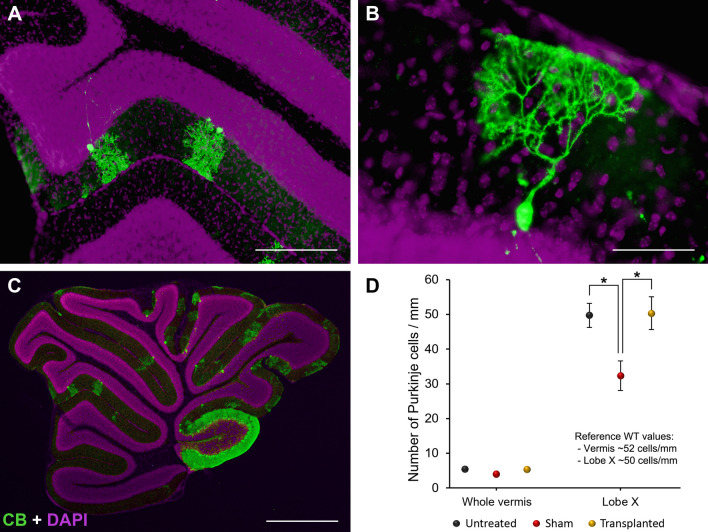
Identification and count of Purkinje cells in the cerebellum of PCD mice at P30. **A**–**C** Immunolabeling of Purkinje cells with CB (green); nuclei were counterstained with DAPI (purple). Note how the surviving Purkinje cells are mainly located in lobe X at P30 (**C**). **D** Chart showing the linear density of Purkinje cells both in the whole vermis and only in lobe X. Note the significant reduction in the density of Purkinje cells in lobe X of sham-operated animals, as well as the neuroprotective effect achieved by transplanting IMCs. Data are expressed as mean ± SEM. *p < 0.05. Scale bar: 200 μm (**A**), 50 μm (**B**) y 1 mm (**C**)

Purkinje cell survival, which is the major population affected in the cerebellum of PCD mice, was also evaluated. To do so, anti-CB antibodies were used to specifically mark Purkinje cells, as previously described [45] (Fig. 6A–C). After estimating the linear density of Purkinje cells in the whole vermis, no differences were detected among the three experimental groups. However, when neuro-resistant lobe X was analyzed separately [41], an increased death of Purkinje cells was detected in sham-operated mice as compared with both untreated (*p* = 0.038) and transplanted animals (*p* = 0.031; Fig. 6D). By contrast, no differences were distinguished between untreated and transplanted mice (*p* = 1.000; Fig. 6D). Therefore, it was found that the surgery in the compromised cerebellum of PCD mice increased the cell death of the surviving Purkinje neurons. Although Purkinje cell survival was the same in the transplanted animals as in untreated PCD mice, these data again highlight that the transplantation of IMCs can reduce the deleterious effects caused by stereotactic surgery, even in a distant region, such as lobe X.

In the supplementary WT mice, three points of analysis were selected, since no especial variations of Purkinje cell density have been described along their cerebellar cortex: lobes IV–V (injection site), lobe VIII (neutral region), and lobe X (neuro-resistant region; 41). The two-way ANOVA test showed no significant differences for either experimental group (*p* = 0.139), region (*p* = 0.848), or interaction (*p* = 0.687; Additional file 1: Fig. S1C). These results are in line with those obtained from the behavioral analysis (see above), as the stereotactic injection did not affect Purkinje cell survival in a healthy cerebellum, independently of the distance from the lesion or the characteristics of the lobe.

### Analysis of microglia

**Fig. 7:**
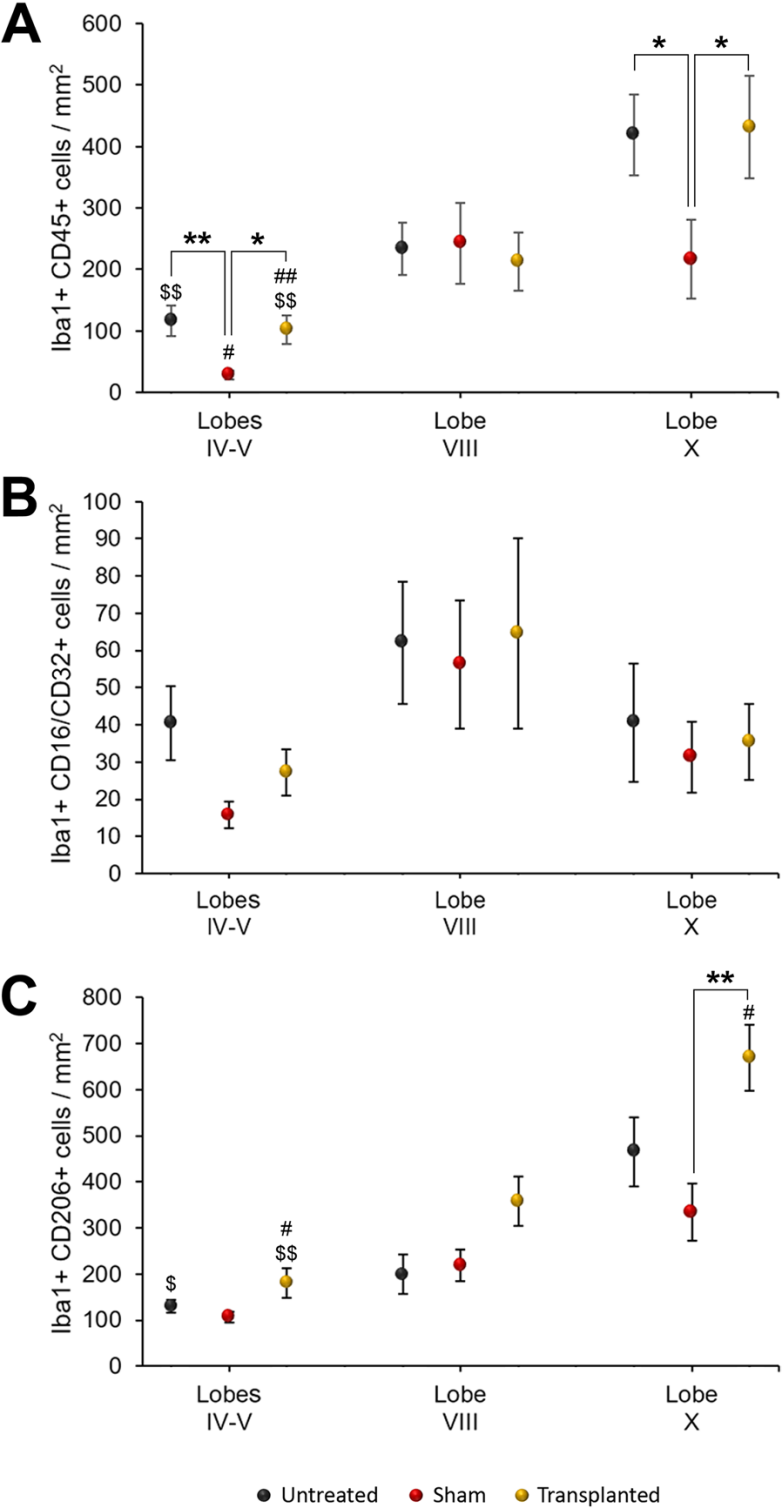
Microglial analysis. Charts showing the density of microglial subpopulations, with either the reactive (Iba1^+^CD45^+^; **A** pro-inflammatory (Iba1^+^CD16/CD32^+^; **B** or anti-inflammatory phenotype (Iba1^+^CD206^+^; **C** Note that surgery reduces the density of reactive microglia, which is restored by IMC transplantation, and the increase in anti-inflammatory neuroprotective microglia. Moreover, both deleterious and neuroprotective effects are focused on lobes IV–V (affected by surgery) and X (neuro-resistant). Data are expressed as mean ± SEM and the statistical analyses were performed using the Ln of represented values for their normalization. For comparing the lobes of all three experimental groups individually: *p < 0.05, **p < 0.01. For comparing the lobes of each experimental group: ^#^*p* < 0.05, ^##^*p* < 0.01, for differences with lobe VIII (neutral); ^$^*p* < 0.05, ^$$^*p* < 0.01, for differences between lobes IV–V and X

As IMCs can modulate neuroinflammation after neural damage [9], we analyzed the different microglial populations of the three experimental groups of PCD mice. The analysis was focused on the molecular and Purkinje cell layers, as these are the layers most affected by the *pcd* mutation [38]. Two-way ANOVA tests revealed significant differences for both reactive and anti-inflammatory microglia (Fig. 7A, C; Additional file 3: Fig. S3) when either the experimental groups (reactive *p* = 0.002; anti-inflammatory *p* = 0.011) or the lobes analyzed were compared (reactive *p* = 0.000; anti-inflammatory *p* = 0.000) without interaction of both factors (reactive *p* = 0.132; anti-inflammatory *p* = 0.773). Conversely, no statistically significant differences were detected for pro-inflammatory microglia, neither comparing the experimental groups (*p* = 0.095) nor the lobes (*p* = 0.315); once more, factors did not show any interaction (*p* = 0.124; Fig. 7B). Therefore, additional statistical analyses were carried out for the two first types of microglial populations.

In general, the reactive microglia of sham PCD mice presented lower densities than untreated PCD mice, with the cell transplant causing standard values to be restored (Additional file 3: Fig. S3A–C). For an in-depth analysis, we compared each lobe separately among the experimental groups. The ANOVA tests revealed differences among treatments for lobes IV–V (injection site; *p* = 0.001) and X (neuro-resistant; *p* = 0.040), but not for lobe VIII (neutral; *p* = 0.978; Fig. 7A). In those lobes with differences, the sham-operated PCD mice presented lower densities of CD45^+^ microglia in comparison with the untreated and transplanted PCD animals, which did not show any differences. The differences detected were more evident in lobes IV–V, where the restrictive Bonferroni’s post hoc tests confirmed they were significant (untreated vs sham, *p* = 0.001; sham vs transplanted,* p* = 0.012; untreated vs transplanted,* p* = 1.000). Then, we compared the density of reactive microglia among the lobes analyzed for each PCD group separately. In this case, ANOVA tests revealed significant differences in the three experimental groups (control, *p* = 0.004; sham, *p* = 0.000; transplanted, *p* = 0.003; Fig. 7A), which were further validated using Bonferroni’s post hoc test. In the case of both untreated and transplanted PCD mice, lower densities of reactive microglia appeared at the injection site (lobes IV–V) and greater densities at the neuro-resistant region (lobe X), with statistically significant differences between them (untreated lobes IV–V vs X, *p* = 0.003; transplanted lobes IV–V vs X, *p* = 0.000; Additional file 3: Fig. S3A–C). The neutral lobe of these PCD groups presented an intermediate density of activated microglial cells, but no other differences were detected with the other lobes. Conversely, in the case of the sham-operated PCD mice, the injected lobes (IV–V) presented lower densities than lobe VIII or X (*p* = 0.000 for both cases) which did not present any differences.

Finally, regarding anti-inflammatory microglia, transplanted PCD mice tended to have higher densities of this cell type than the other experimental groups. To analyze this population, we first compared the lobes of the three experimental groups separately. The ANOVA tests revealed there were differences among these groups but only for neuro-resistant lobe X (*p* = 0.004; Additional file 3: Fig. S3D–F) and not for the others (lobes IV–V, *p* = 0.148; lobe VIII, *p* = 0.276; Fig. 7C). Then, the Bonferroni’s post hoc test confirmed there was a higher density of anti-inflammatory microglia in lobe X of the transplanted animals when compared with the sham-operated animals (*p* = 0.003), with the untreated PCD mice showing intermediate values (Fig. 7C; Additional file 3: Fig. S3D–F). Upon comparing the cerebellar lobes of each experimental group separately, both untreated and transplanted PCD mice presented differences (*p* = 0.005 and *p* = 0.005, respectively), whereas the mere surgery of sham PCD blurred them (sham, *p* = 0.094). In the case of untreated PCD animals, Bonferroni’s post hoc test revealed a higher density of anti-inflammatory microglia in lobe X compared to lobes IV–V and VIII (*p* = 0.009 and *p* = 0.013, respectively; Fig. 7C), which presented similar values. In the case of the transplanted mice, lobes IV–V presented the lowest values of anti-inflammatory microglia, lobe VIII had intermediate values and lobe X had the highest, with all of the differences being significant (Bonferroni’s post hoc test: lobes IV–V vs VIII, *p* = 0.021; lobe VIII vs X, *p* = 0.035; lobes IV–V vs X, *p* = 0.000).

### Study of inflammatory genes

**Fig. 8:**
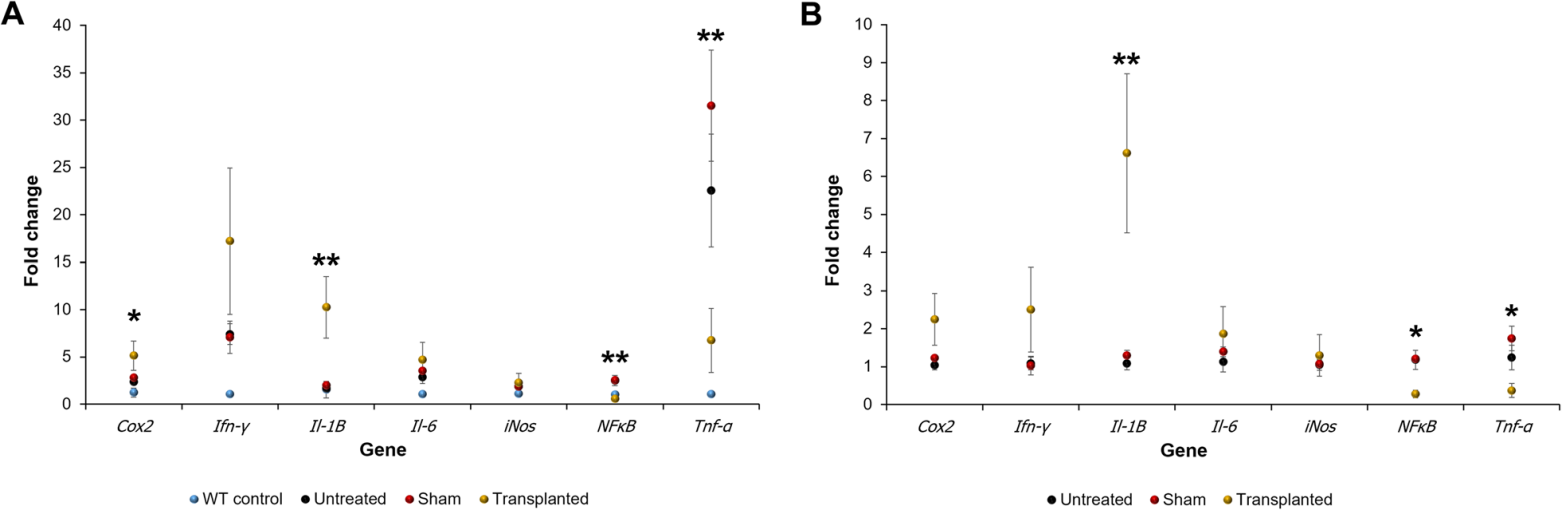
Analysis of the expression of inflammatory genes using qPCR. Charts showing the fold change for inflammation-related genes using either untreated WT (A) or untreated PCD mice (B) as the controls for gene expression. For genes *Cox2* (with only WT as the control, A) and *Il-1β*, only transplanted PCD mice presented a higher expression, with the upregulation of these genes being related to neuroprotection. By contrast, *NfκB* and *Tnf-α* appeared to act as detrimental pro-inflammatory genes, as IMC transplantation reduced their expression, even reaching the levels of the WT control. Data are expressed as mean ± SEM. *p < 0.05, **p < 0.01

As both surgery and transplantation of IMCs changed the densities of microglial populations in PCD mice, the expression of several genes related to inflammation was examined using qPCR. In this case, it is necessary to consider that the cerebellar degeneration of PCD mice also changes the expression of these genes [46]. Therefore, to further investigate the possible variations triggered by either surgery or transplantation, we performed two sets of analyses: [1] comparing the qPCR results of the PCD groups with those of the untreated WT control mice (i.e., whose gene fold change corresponded approximately to 1; Fig. 8A); and [2] comparing only the three experimental PCD groups using untreated PCD mice as the control (i.e., “standard” inflammatory state adjusted to a gene fold change around 1;Fig. 8B).

In the first set of analyses (Fig. 8A), ANOVA tests revealed statistically significant differences for the expression of *Cox2* (*p* = 0.015), *Il-1β* (*p* = 0.002), *NfκB* (*p* = 0.002), and *Tnf-α* (*p* = 0.002). The expression of both *Cox2* and *Il-1β* was higher in transplanted PCD mice as compared with the other three groups. For *Cox2,* Bonferroni’s post hoc test revealed differences for only the WT animals (*p* = 0.012), whereas differences in *Il-1β* were much more evident for the three experimental groups (transplanted PCD vs WT, *p* = 0.005; transplanted PCD vs untreated PCD, *p* = 0.004; transplanted PCD vs sham-operated PCD, *p* = 0.011). In the case of *NfκB* and *Tnf-α*, both untreated and sham-operated PCD mice presented higher expression values than those obtained for transplanted PCD mice and the WT controls. In the case of *NfκB*, Bonferroni’s post hoc tests confirmed the differences found between transplanted PCD mice and the other PCD groups (transplanted PCD vs untreated PCD, *p* = 0.009; transplanted PCD vs sham-operated PCD, *p* = 0.016), and only with sham-operated mice for *Tnf-α* (transplanted PCD vs untreated PCD, *p* = 0.195; transplanted PCD vs sham PCD, *p* = 0.025).

When only PCD mice were analyzed (Fig. 8B), ANOVA tests revealed statistically significant differences for the expression of *Il-1β* (*p* = 0.005), *NfκB* (*p* = 0.014), and *Tnf-α* (*p* = 0.040). In addition, Bonferroni’s post hoc tests confirmed that transplanted PCD mice were always distinct from the other two groups, which did not show differences between them. *Il-1β* was highly expressed in transplanted animals (transplanted PCD vs unharmed PCD, *p* = 0.008; transplanted PCD vs sham PCD, *p* = 0.018), whereas *NfκB* and *Tnf-α* were lowly expressed. For *NfκB*, Bonferroni’s post hoc test validated differences between transplanted mice and the other two PCD groups (transplanted PCD vs untreated PCD, *p* = 0.023; transplanted PCD vs sham PCD, *p* = 0.033), but for *Tnf-α* its expression was only similar to that detected in sham-operated animals, (transplanted PCD vs untreated PCD, *p* = 0.205; transplanted PCD vs sham PCD, *p* = 0.042).

In sum, both untreated and sham-operated PCD mice present a common pattern of gene expression related to inflammation, which seems not to be aggravated by surgery. By contrast, the injection of IMCs changes the expression of some genes, sometimes toward a WT-like expression, but also to an “exaggerated” inflammatory-like state.

## Discussion

The purpose of this study was to investigate whether IMCs isolated from healthy bone marrow can reduce the degeneration/inflammation occurring in the cerebellum of PCD mice, a model of selective degeneration of Purkinje cells. IMCs were transplanted directly into the cerebellum at P20 when neurodegeneration had already begun. The reason this age was selected was that bone-marrow-derived stem cells tend to migrate toward inflamed or damaged areas, a fact that may be due to the effect of chemoattractant signals [47–49]. In addition, we have recently proved that blood cells are specifically attracted to degenerating Purkinje cells [38]. Moreover, both neurodegeneration and inflammation in the central nervous system enhance the regenerative and immunomodulatory properties of several cell types after their transplantation [50–52].

### Monocytic IMCs behave like MDSCs under pathological conditions

Whenever MDSCs are used, their immunosuppressive capability should be checked through functional assays, as this is what distinguishes them from the rest of the myeloid populations [26, 53]. In our case, this is even more relevant, since IMCs were isolated from the bone marrow of WT mice and thus initially lacked immunosuppressive activity [54]. Therefore, we first checked whether IMCs from healthy bone marrow were able to acquire immunosuppressive properties under pathological conditions.

After performing the functional assays previously described, it was confirmed that IMCs were able to suppress the proliferation of activated T cells and promote their apoptosis. Hence, IMCs acquired an immunosuppressive behavior after being cultured in the presence of stimulated T cells, which may be considered similar to the behavior of MDSCs. The fact that IMCs can acquire a potent immunosuppressive capability under pathological conditions eliminates the need for extracting MDSCs from diseased animals, making the use of these cells more amenable for clinical application [33, 34]. To date, several research groups have managed to generate MDSCs after culturing bone-marrow-derived cells, mainly from mice but also from humans [33, 34, 55–57]. Nevertheless, each approach employs a different combination of cytokines during the culturing step, which increases the variability of the immunosuppressive mechanisms of MDSCs generated in this way [57]. Consequently, refining the production of MDSCs in vitro before their clinical application is essential [58]. Importantly, our research lacks this disadvantage, because it contemplates IMC transplantation immediately after their isolation without prior culturing, with the possibility that these cells will acquire the same immunosuppressive capabilities as MDSCs.

### Intracerebellar injection of IMCs avoids the adverse effects provoked by stereotactic surgery

Once characterized, we proceeded to transplant IMCs in PCD mice. In previous studies carried out in our laboratory, we attempted to introduce these cells intravenously, but this route was not favorable for a suitable and consistent arrival into the cerebellum (data not published). In fact, the cell therapy applied for rescuing Purkinje cells from death in PCD mice by intravenous transplantation or bone-marrow replacement has had limited success up until now [4, 5, 48]. That is the reason we decided to inject IMCs directly into the cerebellum, which is what has been performed in the majority of the studies reporting cell therapy in mouse models of cerebellar degeneration [8, 50, 51, 59–67]. The major limitation of using this method is the mechanical injury that causes damage and inflammation to the surrounding tissue [68, 69]. However, this strategy offers several advantages, such as being more direct and achieving greater survival of transplanted cells than those introduced intravenously, although their spreading is rather limited [70, 71]. A large number of GFP^+^ cells were observed at the injection site, lobes IV–V, with an undifferentiated morphology. Although some cells were detected in more distant lobes, such as lobe X, a low level of migration was observed during 10 days of monitorization; instead, the cells remained mostly in lobes IV–V, being detectable in those sections with an evident signal of injection and in the unharmed closest ones. Indeed, it has been reported that cells transplanted directly into the nervous parenchyma hardly move beyond the injection site [71]. Nonetheless, it should be noted that their effects can be spread due to paracrine factors [8, 72], as will be discussed below. The survival rate of transplanted IMCs 10 days after surgery was relatively low (⁓1% of transplanted cells). This result suggests that a relative low number of IMCs can exert a neuroprotective effect even in regions far away of the injection site (i.e., lobe X, see below). In any case, between the time of transplantation and the sacrifice, more cells can be still probably alive at the injection site also exerting their neuroprotective effect.

Regarding the general aspect of the tissue, 10 days after surgery (i.e., at the time of sacrifice at P30), we did not observe any special abnormality in the proximity of the injection site, apart from the gap in the cerebellar cortex caused by the needle. Although we cannot discard some additional damage just before the surgery, it seems that at the timepoint of analysis the cerebellar parenchyma should be recovered from such supplementary tissue injury in both sham and transplanted PCD groups.

When a possible rejection reaction due to transplants was investigated, only a slight increase in the number of T cells was detected in the lobes IV–V of some experimental sham or transplanted PCD subjects. Since this presence of T cells only appears in operated animals, a certain effect of the surgery cannot be discarded, being necessary to bear in mind possible minor infections. In any case, the increase of T cells was always modest, not generalized to all mice that underwent surgery, and did not correlated with either big surgical injuries or elevated numbers of IMCs (Table 3). Moreover, B cells did not show any remarkable increase in any experimental group. Then, we can discard a rejection reaction due to the cell transplant, which can be easily explained by the significant immunosuppressive effect of MDSCs over both activated T and B cells [73].

Concerning the rotarod test, sham-operated mice performed significantly worse as compared with the untreated mice. This motor impairment was not detected in transplanted animals. In addition, these behavioral data correlated with the histological analyses. Thus, a lower density of Purkinje cells was observed in sham-operated mice together with a higher density of apoptotic cells; both phenomena were completely or partially avoided in the transplanted animals. An increased death of Purkinje cells was detected in lobe X, which is relatively distant from the damaged lobe. Therefore, it seems that surgical brain injury was transmitted to other cerebellar regions, probably through glial communication. Although astrocytes and microglia react and convey messages within their immediate environment, they can also communicate at longer distances through diverse signaling molecules [73]. In fact, reactive gliosis has been involved not only in accelerating neurodegenerative processes but also in their distribution [74]. The lack of apparent differences in Purkinje cell density among the PCD groups in the whole vermis could be due to the blurring caused by advanced cerebellar neurodegeneration at P30 [35, 75]. Conversely, lobe X is the only resistant region with a considerable number of surviving Purkinje cells [41], and thus where differences could still be detectable.

As for the supplementary WT mice, increased apoptosis was only detected in the injected lobe and the whole vermis, most probably caused by the aforementioned spreading properties of glial cells [73, 74]. Nevertheless, such an increment of cellular death does not seem particularly relevant, as no other effects of surgery were manifested in relation to Purkinje cell death or their behavior. Therefore, the detrimental effects of a stereotactic injection were only established in already compromised tissue, such as the PCD cerebellum. Given that this type of surgery is usually performed on diseased and not healthy brains, makes the results of this work even more significant.

Taken together, our findings highlight two fundamental facts. The first is that stereotactic surgery exerts detrimental effects on PCD mice at both behavioral and histological levels. This is not surprising, since it has been shown that neurosurgical interventions lead to unavoidable injury to surrounding neural tissue [68, 69], even though techniques have become less invasive, such as stereotactic surgery [76, 77]. In particular, neurosurgery can easily lead to neuroinflammation, bleeding, brain edema, cell death, and eventually cause postoperative neurological deficits [76, 78]. This seems to have exacerbated the inflammation and neuronal death already present in the cerebellum of PCD mice, as well the animals’ decreased motor coordination. Precisely, this is the reason why in previous experiments we initially avoided injecting IMCs directly into brain parenchyma.

The second fact worth noting is that the intracerebellar transplantation of IMCs seems to be a promising therapy that eliminates the previously mentioned adverse effects. Interestingly, to date, such adverse effects are left untreated and are allowed to heal on their own [76]. In fact, there are no specific treatments to prevent surgical brain injury, probably because although an appropriate animal model for studying this issue has not been developed until recently [77]. Given these considerations, it has become a key point in modern neurosurgery to explore new strategies for achieving efficient, safe, and specific neuroprotection [78]. For this reason, the transplantation of IMCs could offer a neuroprotective strategy worth contemplating, especially if we consider that their immunoregulatory potency is acquired and enhanced by inflammation [79]. In addition, it has very recently been shown that MDSCs exert an important immunosuppressive effect over both activated T and B cells [80]. For all these reasons we did not apply any type of immunosuppressive treatment.

### IMCs modulate the inflammatory microenvironment through a neuroprotective effect

The therapeutic benefit induced after the transplantation of several cell types in animal models of cerebellar degeneration seems to be due to the release of neurotrophic and anti-inflammatory factors [8, 59, 72, 81], since both transdifferentiation and cell fusion are infrequent events [7, 8]. Indeed, ongoing clinical trials on the use of bone-marrow cells in treating neurological disorders harness their immunomodulatory and neuroprotective properties [7]. In the case of MDSCs, it is known that they suppress neuroinflammation in diverse models of neurological injury [9]. More specifically, MDSCs can inhibit microglial activation in a model of traumatic brain injury [16], limit the development of inflamed and demyelinated areas in mice with EAE [29, 30, 32], significantly attenuate acute inflammation, and promote tissue repair in a model of spinal cord injury [6]. In this sense, there is an inverse relationship between the number of splenic MDSCs and neuronal damage [82]. Secreted molecules (such as osteopontin) together with infiltrated monocytes can induce neuronal repair in models of advanced neurodegeneration [14, 83, 84]. However, the characterization of such released factors is beyond the scope of this work.

In our specific model (PCD mouse), previous findings have shown an intensified inflammation in the cerebellum of PCD mice, which also impairs the ratio of the different microglial populations [37, 46]. Indeed, the regulation of such inflammation is most likely based on neuroprotection [46]. Here, we have analyzed three common markers for microglial activity: CD45 (reactive), CD16/CD32 (pro-inflammatory), and CD206 (anti-inflammatory). Although the dichotomy of a pro- or anti-inflammatory microglial phenotype can be considered simplistic [85, 86], it has been classically used as it offers a clear idea about the behavior of microglia in the face of specific situations [46, 87]. First, our results have shown there is a reduction in the density of reactive microglia in sham-operated PCD mice, whose values were restored after IMC transplantation. Since activated microglia corresponds with those cells that can exert a neuroprotective effect [88, 89], it is plausible to propose that a certain cell ratio of CD45^+^ would be necessary for protecting against neural damage, beyond the dichotomy of a pro- or anti-inflammatory phenotype [85, 86]. This hypothesis is supported by the fact that the neuro-resistant lobe X [41] presents the highest ratio of CD45^+^ microglial cells in both untreated and transplanted PCD mice. Precisely, the sham-operated animals exhibited a reduced Purkinje cell density in this lobe, where the CD45^+^ microglial cell density was lower than in the other groups.

If we consider the dichotomous pro- or anti-inflammatory phenotype of microglia, the differences detected among the groups were limited only to the anti-inflammatory-like cells. In addition, bearing in mind the simplicity of this classification and the use of only one marker for each phenotype, our results suggest that any treatment, either surgery alone or transplantation, does not specifically change the possible inflammation that can occur in the cerebellar microenvironment of PCD mice. Moreover, the inflammation caused by the massive death of the Purkinje cells of these mice [37] appears so exacerbated that the variation is inappreciable, either positively or negatively, at least in terms of the cellular phenotype (discussed below). In addition, the effects of the procedures and treatments can be manifested through other parameters, such as the previously mentioned glial reaction or the ratio of anti-inflammatory microglia. Regarding the latter cell type, we found that the transplanted animals present, in general, greater cell density than the other PCD mice, a fact that was especially evident in lobe X. These data suggest that the neuroprotective effect of IMCs in terms of cellular immunomodulation is based on an increased number of anti-inflammatory microglial cells. This type of neuroprotection might be specific to severe cerebellar damage, as it has recently been detected in the same region, but in conjunction with another completely different treatment [46]. Furthermore, it has also been ascertained that IMCs can exert direct neuroprotective effects by secreting NGF or osteopontin [14, 90]. In view of this, it is plausible to conclude that IMCs are exerting their effects by modulating their surrounding cells through the release of paracrine factors, although some sort of regulation by cell–cell interactions cannot be dismissed [16, 32].

Finally, the results concerning the expression of inflammatory genes also confirmed that the analysis of inflammation is more complicated than the aforementioned dichotomous classification of microglia. Considering either untreated WT or untreated PCD mice as the controls, three genes showed differences among the experimental groups: *Il-1β*, *NfκB*, and *Tnf-α*. In the case of *NfκB* and *Tnf-α*, a clear neuroprotective effect of the IMC transplantation was detected, as the expression of these inflammatory genes decreased and even reached WT values. Thus, the putative neuronal damage due to exacerbated inflammation was reduced [91, 92]. This effect is especially evident in the case of *Tnf-α*, which presents a 20–30 fold change increase in the untreated and sham-operated PCD mice. In particular, the expression of this gene is important for establishing the pro-inflammatory microglial phenotype [91]. Moreover, the reduction in the expression of the pro-inflammatory genes related to a neuroprotective effect has been previously reported in the same model but with a different treatment [46]. The lack of differences in gene expression between untreated and sham-operated PCD mice corresponds to the results obtained for the CD16/CD32^+^ pro-inflammatory cells, but not the reduced expression found in the transplanted animals. Consequently, the cellular microglial phenotype does not necessarily correspond with a general gene expression, whose origin may reside in other cells apart from microglia. Regarding *Il-1β*, a strong increase in gene expression is detected in transplanted mice in comparison with the other PCD groups and WT mice. This result is noteworthy, as IL-1β is an interleukin clearly related to a pro-inflammatory microglial phenotype [91]. Nevertheless, inflammation should not be always considered as being detrimental, but only if it is exacerbated or prolonged over time [92]. In this sense, the neuroprotective effect of this interleukin may be related to a transitory increase in expression, as previously suggested [46]. In addition, IL-1β is critical for inducing remyelination processes, clearly related to neuroprotection and neural homeostasis [93]. A similar explanation can be applied to the increased expression of *Cox2* in transplanted PCD mice, which has been also previously reported [46]. An increase in *Cox2* mRNA expression was only detected when compared to the WT control. This may suggest that *Cox2* only has a slight influence on the neuroprotective effect of IMCs, as it is somehow increased in all PCD mice.

Even though IMCs could be promoting a neuroprotective microenvironment, it is quite likely that the increased survival rate of Purkinje cells or improved behavior (compared with standard PCD mice) was not achieved because of extremely rapid degeneration. This fact has recently been reported in the case of cell therapy based on healthy bone-marrow-derived cells in PCD mice, which also fails to rescue degenerating Purkinje cells [5]. By contrast, this therapy was associated with neuroprotective effects in the olfactory bulb, another region affected in PCD mice, but with slower progressing degeneration that occurs later on [47].

## Conclusions

In sum, our findings show that monocytic IMCs isolated from healthy bone marrow can acquire immunosuppressive properties, comparable to MDSCs ones, when cultured under pathological conditions. This finding supposes the elimination of the need for extracting MDSCs from diseased subjects, making the use of IMCs much more translational. Furthermore, the intracerebellar transplantation of monocytic IMCs in PCD mice prevents the adverse effects caused by the stereotactic surgery performed, both at the histological and behavioral levels. This phenomenon is extremely interesting in that adequate treatment for damage produced by neurosurgical interventions may result in significant benefits for both patients and clinical practice [77]. Therefore, IMCs could be used as adjuvant agents when performing neurosurgery and thus have useful translational applications. The main limitation of this study is the low survival rate of IMCs after being injected. Although it may suppose a certain advantage (i.e., minimizing the possibility of putative malignant transformations or side effects), certain treatments or surgeries may require prolonged IMCs effect, which is related to their permanence into the injected brain. Then, the possibility of additional IMCs administrations deserves further investigation. Besides, as promising adjuvants, the interaction of IMCs (either synergistic or detrimental) with other specific complementary therapies needs to be assessed. Both research lines are beyond the scope of this work, but they should be addressed in a future to endorse the translational power of IMCs.

Considering the significance of neuroinflammation in the pathophysiology, progression, and outcome of neurodegeneration, our findings offer additional backing for clinical trials designed to evaluate the positive impacts of cell transplantation as a therapeutic strategy for mitigating the inflammatory response in neurodegenerative diseases or neurosurgery and, in this way, try to achieve functional recovery.

### Supplementary Information

**Additional file 1: Figure S1.** Supplementary analyses on WT mice. **A**–**C** Charts showing behavioral and histological variables analyzed in the WT animals, comparing the control (untreated) and sham-operated mice. Note that surgery only increased the density of the apoptotic cells **B** but did not affect motor coordination **A** or the density of Purkinje cells in any of the lobes analyzed (**C**). **C**, **D** Immunofluorescence against calbindin (green) for staining Purkinje cells in the cerebellar sections of the control **D** and sham-operated **E** WT mice; note that surgery does not affect the general density of the neurons apart from the tissue damage at the injection site (arrowheads point to this region in E or its equivalent in untreated animals in **D**. *p < 0.05. Scale bar: 1 mm.

**Additional file 2: Figure S2.** Hematoxylin–eosin staining in sagittal vermis sections corresponding to WT (**A**), PCD (**B**), sham-operated PCD (**C**) and transplanted PCD (**D**) animals. No further signs of the surgery can be appreciated in operated mice **C**, **D** but a damage in the cortex of cerebellar lobes IV–V (asterisks). Note that PCD animals presents a reduced cerebellar size in comparison with WT mice. **E**, **F** Magnification of lesions of C and D. Both cases present a break of the cerebellar cortex structure without apparent qualitative differences, even considering that the extension of lesion in the transplanted mouse **D** is bigger than the corresponding to the sham-operated animal (**C**); some leukocyte-like cells can be appreciated into the edge of the scar in both examples. Transp, transplanted. Scale bar: 1 mm for **A**–**C**, 100 µm for **E**, **F**.

**Additional file 3: Figure S3.** Analysis of microglia. Images of lobe X showing those microglial populations in which differences were detected: CD45^+^ (**A**–**C**, green) and CD206^+^ (**D**–**F**, green). **A**, **D** untreated PCD mice; **B**, **E** sham-operated PCD mice; **C**, **F** transplanted PCD mice. Microglia are labeled with Iba1 (red) and nuclei are counterstained with DAPI (blue). Scale bar: 100 µm.

## Data Availability

The data sets generated and analyzed during this study will be made available by the authors upon reasonable request.

## Abbreviations

CB: Calbindin D-28 k
DAPI: 4′,6-Diamidino-2-phenylindole
EAE: Experimental autoimmune encephalomyelitis
GFP: Green fluorescent protein
IMCs: Immature myeloid cells
MDSCs: Myeloid-derived suppressor cells
PB: Phosphate buffer
PBS: Phosphate buffered saline
PCD: Purkinje Cell Degeneration
RT-qPCR: Real-time quantitative PCR
SEM: Standard error of the mean
TUNEL: Terminal deoxynucleotidyl transferase-mediated dUTP–biotin nick end labeling
WT: Wild type
