# Supplementary figures and images for: Intracerebellar injection of monocytic immature myeloid cells prevents the adverse effects caused by stereotactic surgery in a model of cerebellar neurodegeneration
Source: J Neuroinflammation. 2024 Feb 14;21:49. doi: 10.1186/s12974-023-03000-8 (PMC10867997; doi:10.1186/s12974-023-03000-8)

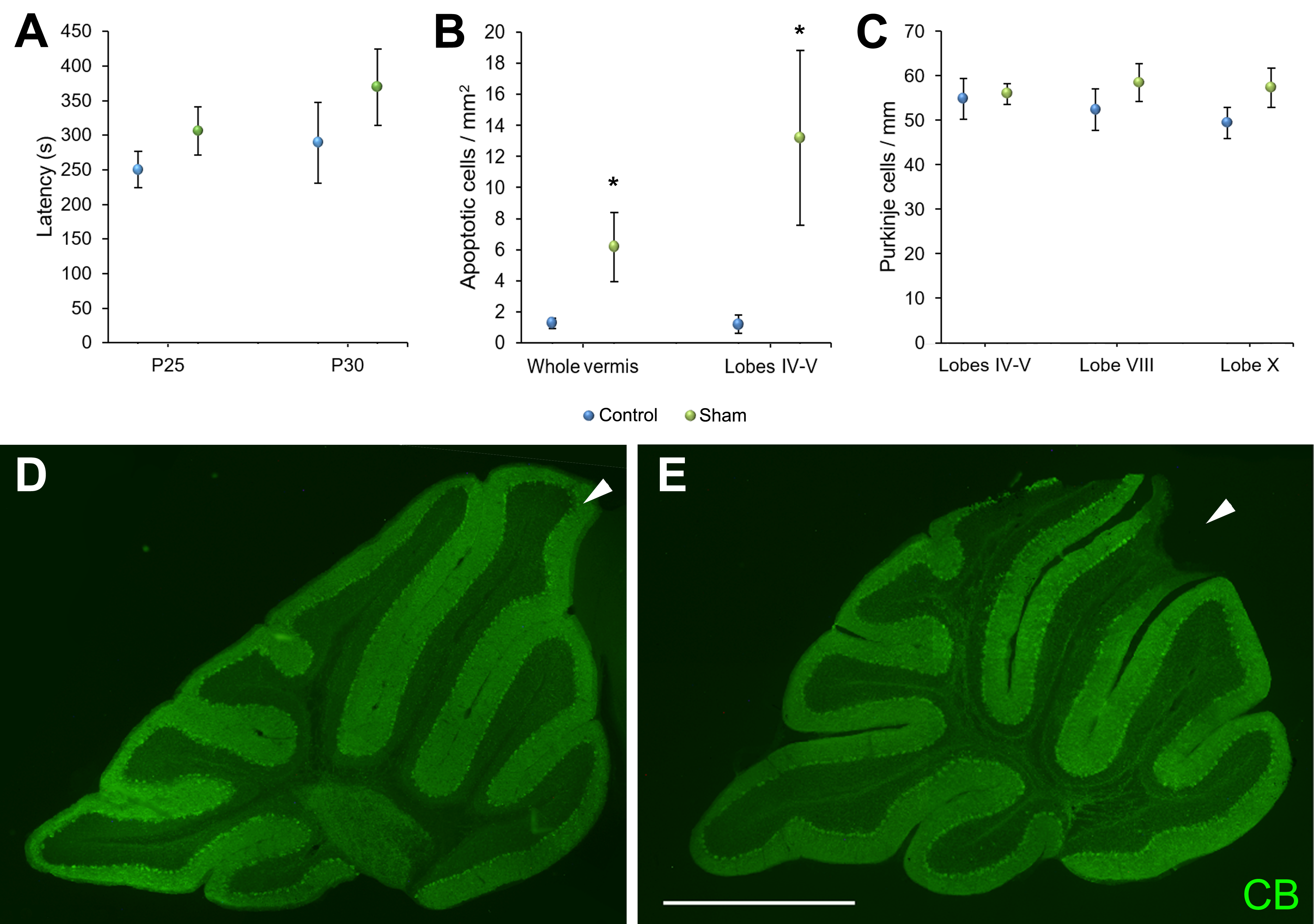

Supplement: Supplementary file 1 — Additional file 1: Figure S1. Supplementary analyses on WT mice. A–C Charts showing behavioral and histological variables analyzed in the WT animals, comparing the control (untreated) and sham-operated mice. Note that surgery only increased the density of the apoptotic cells B but did not affect motor coordination A or the density of Purkinje cells in any of the lobes analyzed (C). C, D Immunofluorescence against calbindin (green) for staining Purkinje cells in the cerebellar sections of the control D and sham-operated E WT mice; note that surgery does not affect the general density of the neurons apart from the tissue damage at the injection site (arrowheads point to this region in E or its equivalent in untreated animals in D. *p < 0.05. Scale bar: 1 mm. [file 12974_2023_3000_MOESM1_ESM.tif]

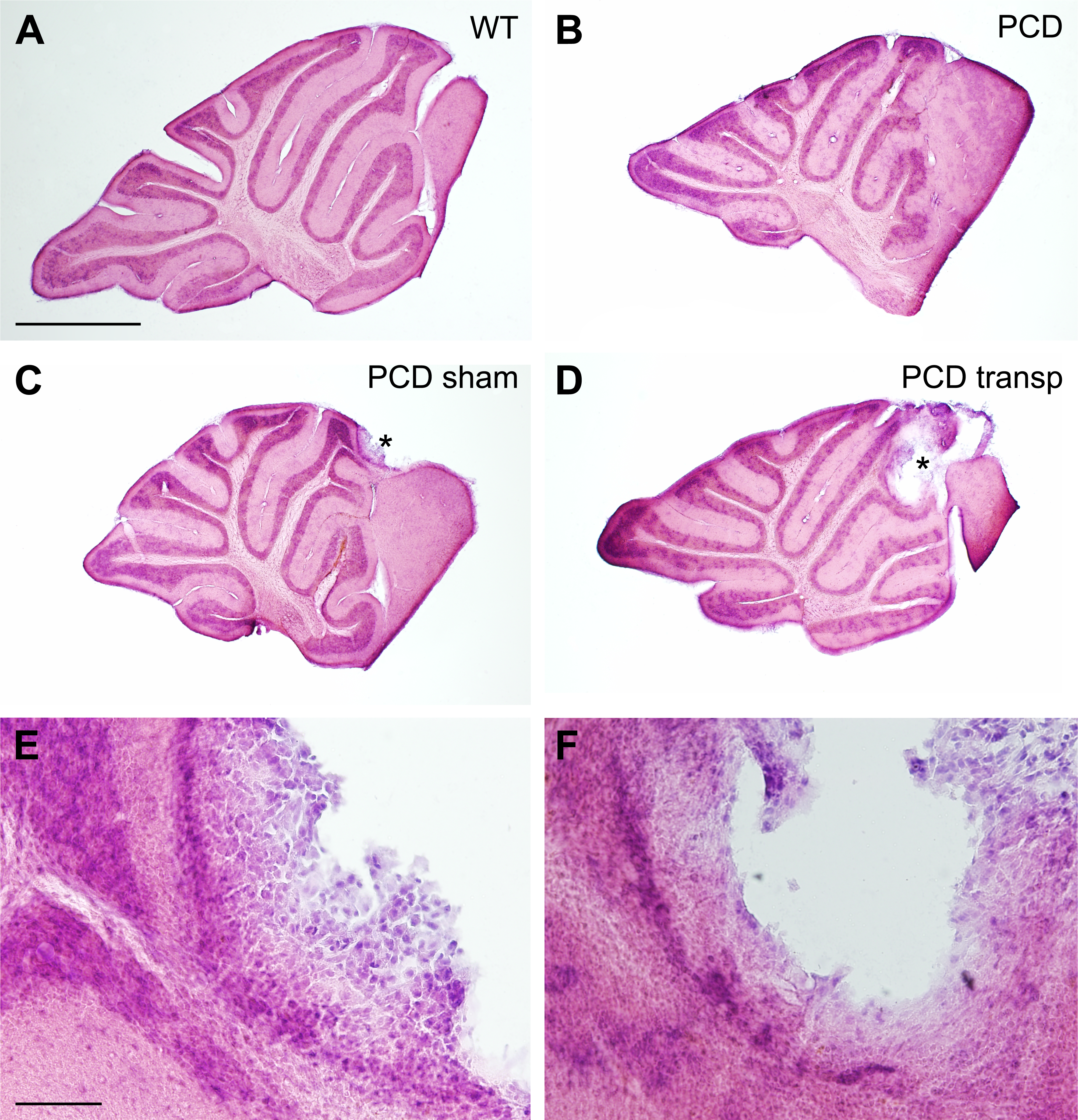

Supplement: Supplementary file 2 — Additional file 2: Figure S2. Hematoxylin–eosin staining in sagittal vermis sections corresponding to WT (A), PCD (B), sham-operated PCD (C) and transplanted PCD (D) animals. No further signs of the surgery can be appreciated in operated mice C, D but a damage in the cortex of cerebellar lobes IV–V (asterisks). Note that PCD animals presents a reduced cerebellar size in comparison with WT mice. E, F Magnification of lesions of C and D. Both cases present a break of the cerebellar cortex structure without apparent qualitative differences, even considering that the extension of lesion in the transplanted mouse D is bigger than the corresponding to the sham-operated animal (C); some leukocyte-like cells can be appreciated into the edge of the scar in both examples. Transp, transplanted. Scale bar: 1 mm for A–C, 100 µm for E, F. [file 12974_2023_3000_MOESM2_ESM.tif]

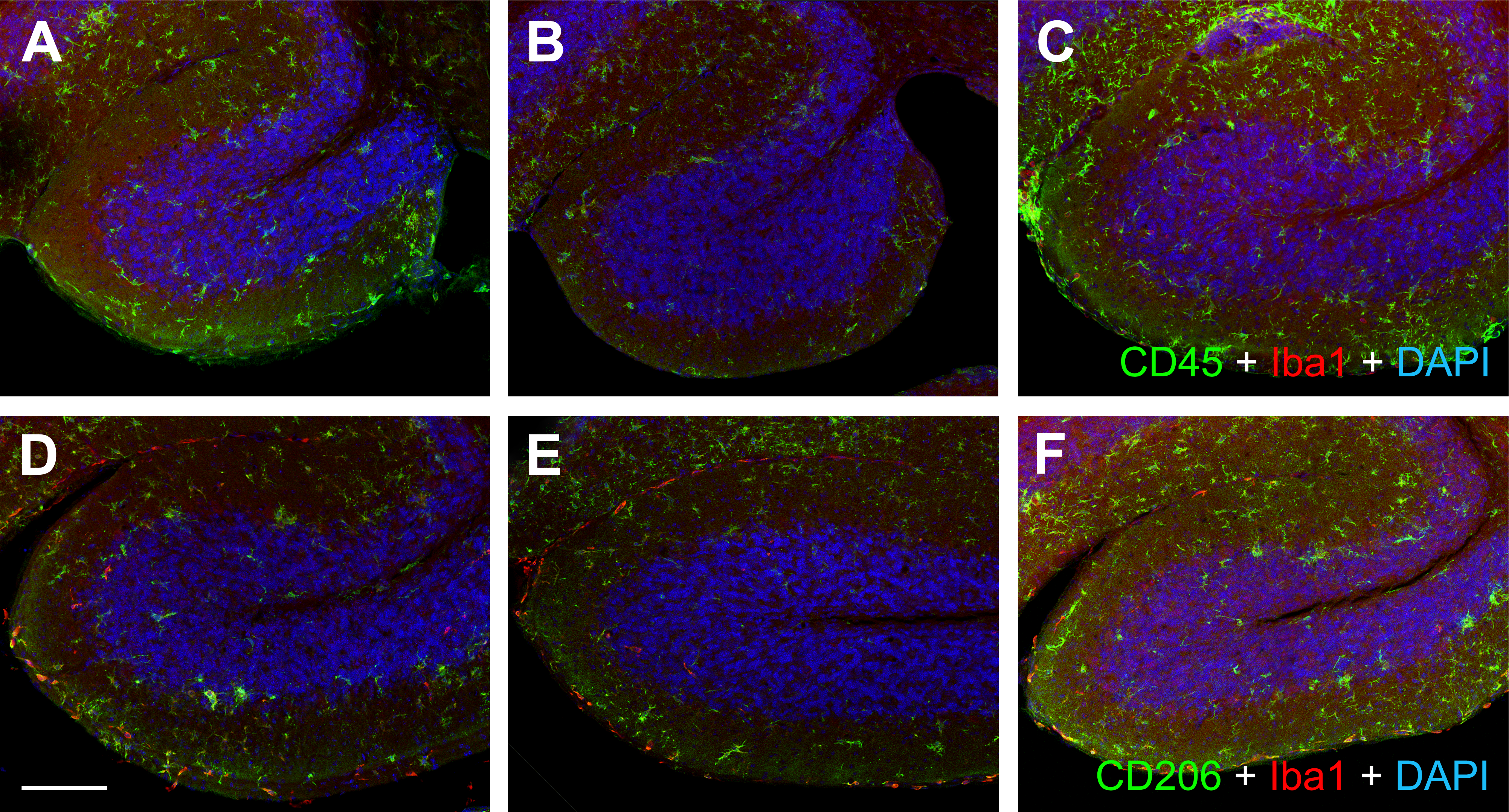

Supplement: Supplementary file 3 — Additional file 3: Figure S3. Analysis of microglia. Images of lobe X showing those microglial populations in which differences were detected: CD45+ (A–C, green) and CD206+ (D–F, green). A, D untreated PCD mice; B, E sham-operated PCD mice; C, F transplanted PCD mice. Microglia are labeled with Iba1 (red) and nuclei are counterstained with DAPI (blue). Scale bar: 100 µm. [file 12974_2023_3000_MOESM3_ESM.tif]
